# Metric analysis of the postcranial skeleton: a comprehensive approach for biological sex estimation in an Italian population

**DOI:** 10.1007/s00414-025-03599-8

**Published:** 2025-10-03

**Authors:** Paolo Morandini, Lucie Biehler-Gomez, Kyra Stull, Cristina Cattaneo

**Affiliations:** 1 LABANOF (Laboratorio di Antropologia e Odontologia Forense), Department of Biomedical Science for Health, University of Milan, Milan, 20133 Italy; 2 Department of Anthropology, University of Nevada, Reno, NV USA

**Keywords:** Forensic anthropology, Sex estimation, Skeletal measurements, Osteometrics, Population-specific standards, Post-cranial metric analysis

## Abstract

**Objectives:**

This paper presents a metric methodology for estimating biological sex specifically tailored to the Italian population. The method considers 121 standard metric measurements derived from 46 bones across various post-cranial regions.

**Materials and methods:**

The sample consists of 400 individuals (M = 200; F = 200) from the 20th century CAL Milano Cemetery Skeletal Collection aged 20 to 104 years old. The sample was divided into a training subset (75%; *n* = 300) and a testing subset (25%, *n* = 100). Intra- and inter-observer analyses, as well as univariate sectioning points, and multivariable logistic regression analyses were performed.

**Results:**

Intra- and inter-observer analysis showed excellent reproducibility of the measurements, with some exceptions generally related to the measurement of long bone diameters. Univariate sectioning points resulted in 18 measurements with accuracies exceeding 90%, and another 48 measurements achieving over 80% accuracy. In total, 43 multivariable logistic regression models were developed for 32 bones, and these models further increased the accuracy.

**Discussion:**

The validation of these models demonstrated that the proposed methodology allows for sex estimation with accuracies of over or near 90% and minimal class discrimination bias across all post-cranial skeletal regions. The highest accuracies – with both sectioning points and multivariable models – were the radius (96.8%), scapula (95.3%), and tibia (95.2%). This study introduces a comprehensive metric standard for the Italian population and highlights the accuracy of the metric approach for estimating biological sex.

**Supplementary Information:**

The online version contains supplementary material available at 10.1007/s00414-025-03599-8.

## Introduction

Estimating biological sex (understood here as estimated assigned sex at birth) is a fundamental step in anthropological studies, both in forensic and bioarchaeological contexts. The metric method stands out for its increased objectivity and higher intra- and inter-observer agreement [1, 2]. Male skeletal dimensions are on average 8–20% greater than those of females [3, 4], depending on the populations and characteristics considered, making metric traits valid for sex estimation. Particularly, from Pearson’s pioneering studies, postcranial measurements have captured the attention of anthropologists, proving to be more accurate than cranial metric and morphological methods [2]. Numerous studies have developed and refined metric methods for sex estimation based on various anatomical regions of the postcranium [2, 5, 6]. Most metric studies focus on the long bones of the upper and lower limbs, the shoulder girdle, and the pelvis—regions that frequently yield measurements with a high degree of sexual dimorphism [2, 5–12]. However, other studies have reported excellent potential for sex estimation from measurements of less commonly considered anatomical areas, such as the vertebral column [13–15], thorax [16–19], and carpal [20–22] and tarsal bones [23, 24].

A limitation of metric approaches is the interpopulation variability. Although common patterns exist in sexual dimorphism across different populations, genetic, environmental, and cultural differences can significantly influence skeletal dimensions [25, 26]. These factors result in varying dimensions and degrees of sexual dimorphism among populations necessitating the development of population-specific methods. Applying methods developed for one population to another can lead to a significant loss of accuracy [5]. Some attempts have been made to develop universally applicable methods, such as those proposed by Albanese, who argues that metric approaches can be effective across populations when certain criteria are met: the use of a strategically chosen reference sample representing diverse degrees of human variation, the application of a robust alternative statistical framework, and the identification of meaningful and reproducible combinations of sexually dimorphic measurements [27, 28]. However, it is well-established that the use of population-specific references significantly improves the accuracy of sex estimation [2, 5, 11]. Regarding the Italian population, only a few studies have provided adequate standards for certain body regions [11, 29–33], but their applicability is limited as they focus on a restricted number of skeletal elements and often rely on small sample sizes.

This paper proposes a metric approach based on the postcranial skeleton specific to the Italian population. This approach seeks to facilitate application in many different contexts by using many post-cranial regions, and mitigate the effects of preservation, which can compromise the integrity of morphologically diagnostic anatomical parts, applicable to both individual remains and contexts involving commingled remains. Univariate analyses with sectioning points as well as multivariable analyses using logistic regression and all bones are employed to enhance estimation accuracy and reveal the metric variables that lead to the highest accuracies.

## Materials and methods

**Fig. 1:**
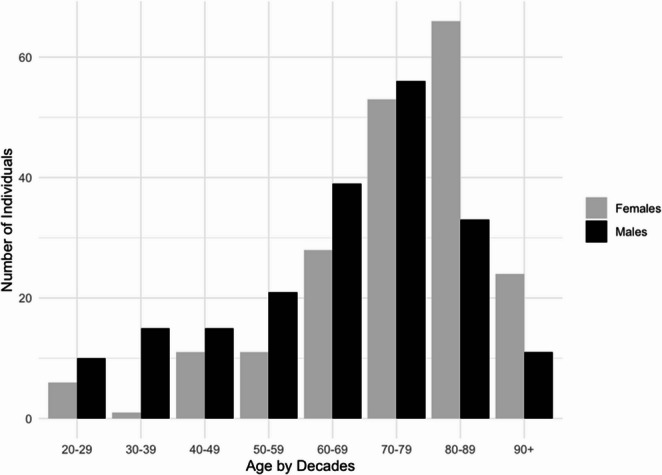
Sample distribution by biological sex and age group

The sample consisted of 400 skeletons, with equal representation and distribution of the sexes (200 males and 200 females). Individuals’ ages at death ranged from 20 to 104 years, with a mean age-at-death of 66 years (standard deviation [SD] = 18; range 20–101) for males and 75 years (SD = 16; range 21–104) for females (Fig. 1). The sample originated from the Milano Cemetery Skeletal Collection, which is part of the Laboratory of Forensic Anthropology and Odontology (LABANOF) Anthropological Collection (CAL). This is a contemporary and documented osteological collection consisting of unclaimed skeletons from Milanese cemeteries [34]. The agreement between Milanese cemeteries and the LABANOF for the recovery of unclaimed skeletal remains for educational and scientific research purposes is regulated by Article 43 of the Mortuary Police Regulation (Decree of the President of the Republic No. 285 of 10/09/1990). Documentation for each individual was possible because of a collaboration with the Local Health Authority (ASL). Thus, the selected individuals have known biological sex, age-at-death, and have birth dates ranging from 1880 to 1972 and death dates from 1927 to 2001.

In total, 121 measurements derived from 46 postcranial bones were investigated (Supplementary Material table A). Measurements were selected among the most representative and accurate according to existing literature, principally based on Langley et al. 2016 [35] and implemented to cover all post-cranial body regions. For bilateral measurements, the left side of the body was considered, with the right side measured in case the left was absent. The number of measurements taken for each skeleton was limited by its state of preservation. Consequently, if a bone was absent or if taphonomic alterations were present at the reference points, measurement was not possible. Additionally, measurements were not recorded in cases where pathological signs and bone calluses altered the original anatomical configuration. Measurements were taken using a digital caliper with a measurement precision of 0.1 mm or an osteometric board. Circumferential measurements were obtained using a flexible tape measure. All measurements were taken by the first author of the article (PM).

### Statistical analyses

For the intra-observer analysis, 17 individuals were randomly selected, and measurements were collected by the author (PM) about six months after the initial data collection. For the inter-observer analysis, 15 skeletons were randomly selected, and measurements were performed by two of the authors (PM and LB-G). For these analyses, measurements were taken on both sides of the body. Intra- and inter-observer analyses involved calculating the technical error of measurement (TEM), relative TEM (rTEM) [36], and reliability coefficient (R). Acceptable values for rTEM are based on existing literature using the same measurements, with an intra-observer error set at < 1.5% and an inter-observer error at < 2.0% [37]. These thresholds were adopted to ensure stricter standards of measurement precision. However, some studies report that rTEM values up to < 5% can still be considered acceptable [e.g. 38, 39]. The reliability coefficient R measures the consistency of repeated measurements, both by the same observer (intra-observer error) and between different observers (inter-observer error). The range of R is from 0 (not reliable) to 1 (perfectly reliable). A reliability greater than 0.95 is considered acceptable in the literature [40].

Descriptive statistics were performed using Microsoft Excel^®^ and software JASP^®^ (version 0.18.3) and were calculated for each variable. Independent Student’s t-tests were used to assess differences in dimensions between male and female measurements when data were normally distributed, while Mann-Whitney U tests were applied for non-parametric data (significance at *p* < 0.05). Data normality was evaluated beforehand using the Shapiro-Wilk test.

The sample was randomly divided into a training subset, comprising 75% of the individuals (300 skeletons, 150 females/150 males), and a test subset, consisting of the remaining 25% of the individuals (100 skeletons, 50 females/50 males). The data partitioning was carried out using R^®^ statistical software and the caret package [41] (version 4.4.0). The training sample was used to develop the univariate sectioning points and multivariable logistic regression analyses, while the test sample was utilized to validate the derived models. The sectioning points were obtained by averaging the mean values for males and females. Measurements higher than the sectioning point are classified as male, those lower are classified as female, and values equal to the sectioning point are categorized as indeterminate. The training and testing sample were used to evaluate the performance of the sectioning points. The accuracy percentages for each measurement were calculated by dividing the correct number for each sex by the total number of individuals for that sex, and then averaging the sex-specific classification rates to generate overall classification rates. Additionally, class discrimination bias was calculated by subtracting the female correct classifications from the male correct classifications. Class discrimination bias values between − 5% and + 5% are generally recommended in forensic contexts [42].

Regarding multivariable analysis, logistic regression equations were developed bone by bone. Logistic regression models were generated on the training sample and required all individuals to have all measurements per bone, which means each bone has a different sample size dependent on measurement availability. Furthermore, for some bones, additional logistic regression models were included to also consider reliability and applicability of the selected variables. The general form of the logistic regression equation is expressed as:

*p* = 1/(1 + e^−Z^).

where 𝑝 represents the probability of the outcome (in this case, male or not male), ‘e’ is Euler’s constant (e ≈ 2.71828) and 𝑍 is the linear combination of the independent variables (calculated by multiplying each measurement by its corresponding coefficient and adding the intercept). The equation produces a value between 0 and 1. A result greater than 0.5 indicates a male classification, while a result below 0.5 suggests a female classification. This value also reflects the likelihood that the observed measurements correspond to a male, with the female probability being 1-𝑝. The testing set was used to validate the models generated with the training set. The accuracy rates achieved by the testing sets are used for all further interpretations and what practitioners should also report when using these methods.

## Results

### Intra- and inter- observer agreement

Results of the intra- and inter-observer analyses are summarized in Table 1 and include the TEM, rTEM, and the reliability coefficient (R). Regarding intra-observer error, seven measurements exhibit a rTEM > 1.5%, though the reliability coefficient indicated an almost perfect correlation for the seven measurements. In fact, all measurements were found to be acceptable according to the reliability of coefficient standards (> 0.95).

**Table 1:** Technical error of measurement (TEM), relative technical error of measurement (rTEM) and coefficient of reliability (R) values for inter- and intra-observer error tests. N is the number of observations per test. rTEM exceeding standard thresholds are in bold

	Bone	Measurement	Intra-observer				Inter-observer			
			*n*	TEM	rTEM	*R*	*n*	TEM	rTEM	*R*
V1	CLAVICLE	Max. length	23	0.580	0.39%	0.997	16	0.608	0.42%	0.998
V2	CLAVICLE	Sagittal diameter MS	30	0.362	**3.20%**	0.980	28	0.425	**3.68%**	0.975
V3	CLAVICLE	Vertical diameter MS	30	0.318	**3.10%**	0.975	28	0.395	**3.89%**	0.945
V4	SCAPULA	Height	24	0.757	0.49%	0.997	6	0.289	0.18%	0.998
V5	SCAPULA	Medio-lateral breadth	29	0.315	0.29%	0.983	14	0.523	0.49%	0.996
V6	SCAPULA	Glen. cavity height	33	0.375	1.03%	0.990	19	0.451	1.27%	0.981
V7	SCAPULA	Glen. cavity breadth	32	0.203	0.76%	0.994	20	0.400	1.52%	0.983
V8	HUMERUS	Epicondylar breadth	31	0.136	0.23%	1.000	23	0.215	0.36%	0.998
V9	HUMERUS	Max. head diameter	27	0.161	0.36%	0.998	25	0.272	0.60%	0.996
V10	HUMERUS	Sagittal diameter MS	33	0.273	1.32%	0.992	27	0.515	**2.49%**	0.972
V11	HUMERUS	Transverse diameter MS	33	0.336	**1.75%**	0.975	27	0.495	**2.48%**	0.980
V12	HUMERUS	Max. length	31	0.238	0.08%	1.000	24	0.722	0.22%	0.999
V13	ULNA	Max. length	23	0.304	0.12%	1.000	14	0.681	0.27%	0.999
V14	ULNA	Physiological length	26	0.286	0.13%	1.000	15	0.483	0.22%	0.999
V15	ULNA	Min. circumference	26	0.360	1.00%	0.995	17	1.485	**4.22%**	0.959
V16	ULNA	Max. diameter MS	31	0.197	1.22%	0.992	19	0.493	**3.06%**	0.968
V17	ULNA	Min. diameter MS	31	0.177	1.53%	0.992	19	0.461	**3.94%**	0.957
V18	ULNA	Trochlear notch breadth	32	0.241	1.21%	0.992	23	0.280	1.38%	0.983
V19	RADIUS	Max. length	27	0.379	0.16%	1.000	16	0.637	0.27%	0.999
V20	RADIUS	Sag. diameter MS	30	0.206	**1.79%**	0.981	21	0.299	**2.61%**	0.963
V21	RADIUS	Trans. diameter MS	30	0.160	1.06%	0.993	21	0.313	**2.09%**	0.980
V22	RADIUS	Max. head diameter	28	0.142	0.65%	0.996	10	0.132	0.58%	0.988
V23	SCAPHOID	Max. length	11	0.083	0.30%	0.999	7	0.158	0.61%	0.998
V24	SCAPHOID	Max. width	11	0.242	1.47%	0.983	7	0.177	1.09%	0.994
V25	LUNATE	Length	11	0.169	0.96%	0.992	7	0.275	1.59%	0.989
V26	LUNATE	Width	11	0.176	0.97%	0.993	7	0.189	1.10%	0.995
V27	TRIQUETRAL	Max width	7	0.200	1.34%	0.989	2	0.100	0.79%	1.000
V28	TRIQUETRAL	Max height	7	0.180	1.12%	0.999	2	0.224	1.41%	1.000
V29	PISIFORM	Max. length	3	0.163	1.05%	0.991	1	0.000	0.00%	-
V30	PISIFORM	Max. width	3	0.224	**2.21%**	1.000	1	0.000	0.00%	-
V31	TRAPEZIUM	Max. length	10	0.032	0.13%	1.000	5	0.055	0.24%	0.999
V32	TRAPEZIUM	Height	10	0.114	0.60%	0.997	5	0.167	1.01%	0.998
V33	TRAPEZOID	Length of palmar surf.	10	0.059	0.34%	0.999	5	0.100	0.62%	0.963
V34	TRAPEZOID	Width of dorsal surf.	10	0.124	1.04%	0.996	5	0.161	1.45%	0.995
V35	CAPITATE	Height	11	0.193	0.80%	0.989	12	0.110	0.47%	0.999
V36	CAPITATE	Width of distal base	11	0.211	1.52%	0.992	10	0.248	1.87%	0.993
V37	HAMATE	Max. height	14	0.305	1.28%	0.989	5	0.319	1.37%	0.970
V38	HAMATE	Max. width	15	0.161	0.77%	0.997	6	0.150	0.72%	0.996
V39	MC1	Max. length	14	0.100	0.21%	1.000	11	0.350	0.78%	0.997
V40	MC2	Max. length	17	0.153	0.22%	0.999	13	0.302	0.46%	0.998
V41	MC3	Max. length	19	0.218	0.31%	0.998	12	0.456	0.71%	0.992
V42	MC4	Max. length	16	0.132	0.23%	0.999	13	0.297	0.54%	0.994
V43	MC5	Max. length	16	0.192	0.35%	0.999	11	0.334	0.64%	0.989
V44	STERNUM	Manubrium length	11	0.286	0.57%	0.999	5	0.615	1.21%	0.953
V45	STERNUM	Body length	12	0.423	0.45%	0.999	7	0.308	0.32%	1.000
V46	STERNUM	Total length	8	0.336	0.24%	1.000	3	0.939	0.63%	0.999
V47	STERNUM	Manubrium max. width	8	1.347	**2.32%**	0.975	4	0.600	1.03%	0.990
V48	STERNUM	Sup. body width	13	0.179	0.68%	0.999	7	0.179	0.66%	0.998
V49	STERNUM	Inf. Body width	11	0.355	0.98%	0.999	5	0.363	1.13%	0.996
V50	1^ST^ RIB	Max. chord	20	1.001	1.18%	0.991	8	1.186	1.48%	0.996
V51	1^ST^ RIB	Min. chord	20	0.895	**1.66%**	0.991	12	1.349	**2.54%**	0.985
V52	4^TH^ RIB	Width	11	0.143	0.86%	0.999	3	0.261	**2.08%**	0.995
V53	ATLAS	Sagittal diameter	12	0.168	0.36%	0.999	9	0.355	0.77%	0.987
V54	ATLAS	Transverse diameter	11	0.456	0.59%	0.993	4	0.146	0.18%	0.998
V55	C2	Max. sagittal length	9	0.399	0.80%	0.976	6	0.597	1.26%	0.981
V56	C2	Max. height	10	0.317	0.81%	0.961	10	0.404	1.06%	0.969
V57	C2	Max. breadth sup. facets	11	0.060	0.13%	1.000	10	0.460	1.01%	0.994
V58	C7	Ant. body height	9	0.082	0.61%	0.997	10	0.221	1.62%	0.991
V59	C7	Sag. length	8	0.139	0.22%	1.000	8	0.255	0.42%	0.998
V60	C7	Max width	6	0.116	0.17%	1.000	1	0.566	0.76%	-
V61	T1	Ant. body height	9	0.155	1.04%	0.995	10	0.306	1.96%	0.991
V62	T1	Sag. length	6	0.272	0.43%	0.966	5	0.672	1.07%	0.985
V63	T1	Width at costal head facets	9	0.248	0.73%	0.998	10	0.378	1.14%	0.990
V64	T12	Ant. body height	15	0.197	0.82%	0.985	6	0.132	0.60%	0.999
V65	T12	Sag. length	10	0.546	0.76%	0.993	3	0.551	0.72%	0.999
V66	T12	Width at costal head facets	11	0.102	0.24%	1.000	6	0.484	1.12%	0.989
V67	L1	Ant. body height	13	0.182	0.71%	0.990	8	0.251	1.01%	0.989
V68	L1	Sag. length	8	0.322	0.41%	0.998	2	0.400	0.55%	1.000
V69	L1	Max. endplate width	11	0.207	0.45%	0.999	8	0.605	1.37%	0.986
V70	L5	Ant. body height	10	0.192	0.67%	0.993	5	0.210	0.74%	0.996
V71	L5	Sag. length	6	0.198	0.26%	0.999	5	0.724	0.98%	0.997
V72	L5	Max. endplate width	9	0.071	0.14%	1.000	6	0.765	1.56%	0.977
V73	OS COXAE	Max. heigth	26	0.398	0.19%	0.999	20	0.791	0.38%	0.999
V74	OS COXAE	Min. ischium length	26	0.474	0.86%	0.992	17	0.529	0.96%	0.993
V75	OS COXAE	Iliac breadth	18	0.656	0.42%	0.987	13	1.268	0.83%	0.967
V76	OS COXAE	Min. pubis length	16	0.619	0.86%	0.991	10	0.830	1.19%	0.968
V77	OS COXAE	Max. I.P. ramus length	18	0.861	0.89%	0.986	14	1.057	1.09%	0.929
V78	SACRUM	S1 trans. diameter	15	0.568	1.20%	0.992	11	0.486	1.07%	0.994
V79	SACRUM	S1 sagittal diameter	14	0.210	0.67%	0.996	10	0.518	1.74%	0.978
V80	SACRUM	Anterior height	12	0.294	0.28%	0.999	4	0.898	0.88%	0.992
V81	SACRUM	Anterior breadth	13	1.615	1.47%	0.953	10	1.194	1.11%	0.979
V82	FEMUR	Epicondylar breadth	29	0.256	0.32%	0.998	23	0.336	0.42%	0.998
V83	FEMUR	Max. head diameter	31	0.209	0.46%	0.998	25	0.340	0.74%	0.994
V84	FEMUR	Circumference MS	34	1.007	1.18%	0.991	27	1.018	1.17%	0.986
V85	FEMUR	Trans. Diameter MS	34	0.269	1.03%	0.992	27	0.214	0.82%	0.992
V86	FEMUR	Sagittal diameter MS	34	0.414	1.47%	0.983	27	0.395	1.40%	0.989
V87	FEMUR	Trans. subtroch. diameter	34	0.404	1.36%	0.987	27	0.542	1.71%	0.959
V88	FEMUR	Bicondylar length	32	0.534	0.12%	1.000	26	0.858	0.19%	0.999
V89	FEMUR	Max. length	32	0.519	0.12%	1.000	26	0.888	0.20%	0.999
V90	FEMUR	Med. cond. max. length	27	0.410	0.66%	0.996	23	0.509	0.82%	0.992
V91	FEMUR	Lat. cond. max. length	26	0.258	0.42%	0.997	25	0.561	0.91%	0.988
V92	TIBIA	Prox. epiphyseal breadth	22	0.411	0.56%	0.999	21	0.504	0.69%	0.995
V93	TIBIA	Dist. epiphyseal breadth	25	0.447	0.99%	0.991	17	0.875	1.90%	0.983
V94	TIBIA	Nut. for. circumference	30	0.775	0.86%	0.995	27	1.421	1.55%	0.984
V95	TIBIA	Nut. for. trans. diameter	30	0.373	1.51%	0.988	27	0.495	2.00%	0.981
V96	TIBIA	Nut. for. AP diameter	30	0.483	1.51%	0.981	27	0.558	1.77%	0.981
V97	TIBIA	Length	29	0.420	0.12%	1.000	25	0.721	0.21%	0.999
V98	FIBULA	Max. diameter MS	32	0.163	1.05%	0.995	25	0.316	**2.13%**	0.968
V99	FIBULA	Max. length	20	0.237	0.07%	1.000	16	0.530	0.15%	1.000
V100	CALCANEUS	Max. length	18	0.324	0.40%	0.997	17	0.514	0.64%	0.991
V101	CALCANEUS	Middle breadth	20	0.352	0.85%	0.993	20	0.737	1.81%	0.966
V102	TALUS	Length	21	0.186	0.32%	0.999	23	0.469	0.80%	0.994
V103	TALUS	Breadth	21	0.370	0.92%	0.994	21	0.319	0.76%	0.993
V104	CUBOID	Length	17	0.140	0.37%	0.998	18	0.315	0.83%	0.992
V105	CUBOID	Breadth	16	0.217	0.77%	0.997	14	0.342	1.23%	0.976
V106	NAVICULAR	Length	19	0.267	1.28%	0.970	18	0.433	2.02%	0.971
V107	NAVICULAR	Breadth	19	0.266	0.66%	0.993	18	0.327	0.81%	0.983
V108	MED CUNEIFORM	Length	19	0.162	0.61%	0.992	17	0.459	1.71%	0.970
V109	MED CUNEIFORM	Height	19	0.326	0.98%	0.988	17	0.308	0.94%	0.988
V110	INT CUNEIFORM	Length	16	0.262	1.39%	0.972	16	0.341	1.81%	0.980
V111	INT CUNEIFORM	Height	16	0.198	0.90%	0.994	13	0.197	0.93%	0.989
V112	LAT CUNEIFORM	Length	15	0.108	0.45%	0.995	18	0.376	1.50%	0.964
V113	LAT CUNEIFORM	Height	15	0.211	0.92%	0.992	14	0.212	0.93%	0.976
V114	MT1	Max. length	17	0.362	0.58%	0.993	10	0.209	0.33%	0.998
V115	MT2	Max. length	19	0.143	0.19%	0.999	15	0.289	0.40%	0.998
V116	MT3	Max. length	17	0.193	0.27%	0.999	14	0.235	0.35%	0.998
V117	MT4	Max. length	16	0.203	0.29%	0.999	14	0.268	0.40%	0.999
V118	MT5	Max. length	18	0.292	0.42%	0.996	11	0.491	0.71%	0.963
V119	PATELLA	Max. length	8	0.277	0.63%	0.993	10	0.366	0.90%	0.988
V120	PATELLA	Max. breadth	7	0.125	0.28%	0.999	10	0.285	0.67%	0.995
V121	PATELLA	Max. thickness	8	0.224	1.08%	0.983	14	0.244	1.21%	0.985

Concerning inter-observer analysis, a rTEM > 2.0% was found for 13 measurements, yet like the intra-observer analyses, all measurements met the reliability coefficient standards (*R* > 0.95), indicating a strong association between the measurements taken by the two observers. The measurements that showed a higher-than-acceptable intra-observer error also exhibited an inter-observer error beyond the acceptable standards, with the notable exception of the maximum width of the manubrium, which only displayed an intra-observer error.

No measurement was excluded from the evaluation based on intra- and interobserver error. Moreover, although we adopted stricter thresholds regarding the rTEM (< 1.5% and < 2.0%), it is important to note that all measurements also fall within the broader limits (< 5%) considered acceptable in the literature. Nonetheless, it is recommended to exercise caution when measuring those variables that may be subject to lower reliability.

### Differences between sexes

The average male measurements were found to be greater than those of females, except for three measurements: minimum pubic length, maximum ischiopubic ramus length, and anterior width of the sacrum (Table 2). Most measurements showed a significant *p*-value, less than 0.001, indicating a strong difference between the measurements in the two sexes. Three measurements were not statistically significant: minimum pubic length (*p* = 0.286), maximum ischiopubic ramus length (*p* = 0.101) and anterior width of the sacrum (*p* = 0.832). Consequently, these measurements were excluded from single variable analyses.

**Table 2:** Results of the single variable analysis. Sectioning points (sec point), in millimeters, along with their respective accuracy and class discrimination bias (CD bias), are provided. M, F, and T indicate the number of observations for males, females, and total, respectively. NS results are non-significant

	Bone	Measurement	Training sample								Test sample				
			M	F	T	mean M	mean F	sec point	accuracy	CD bias	M	F	T	accuracy	CD bias
V1	CLAVICLE	Max. length	119	94	213	154.0	139.0	146.5	82.6%	−6.3%	32	32	64	89.1%	−9.4%
V2	CLAVICLE	Sagittal diameter MS	139	131	270	12.2	10.1	11.2	81.9%	−2.6%	45	47	92	85.9%	−2.8%
V3	CLAVICLE	Vertical diameter MS	139	130	269	10.9	8.8	9.9	84.0%	−1.2%	45	48	93	76.3%	2.8%
V4	SCAPULA	Height	80	50	130	159.8	138.0	148.9	84.6%	−2.3%	28	18	46	89.1%	−8.7%
V5	SCAPULA	Medio-lateral breadth	103	73	176	110.7	98.3	104.5	88.1%	−6.3%	35	28	63	88.9%	−7.1%
V6	SCAPULA	Glen. cavity height	140	116	256	38.4	33.2	35.8	90.2%	1.1%	43	44	87	95.4%	−4.7%
V7	SCAPULA	Glen. cavity breadth	134	116	250	28.8	24.6	26.7	85.2%	1.3%	41	43	84	91.7%	2.0%
V8	HUMERUS	Epicondylar breadth	136	117	253	62.5	53.9	58.2	89.7%	−3.2%	42	49	91	91.2%	−1.4%
V9	HUMERUS	Max. head diameter	133	108	241	47.7	41.8	44.7	89.6%	−2.0%	45	39	84	89.3%	−10.4%
V10	HUMERUS	Sagittal diameter MS	143	141	284	21.5	18.5	20.0	82.0%	2.4%	47	51	98	71.4%	5.8%
V11	HUMERUS	Transverse diameter MS	143	141	284	20.5	17.3	18.9	81.7%	−1.2%	47	51	98	81.6%	2.6%
V12	HUMERUS	Max. length	133	118	251	324.5	295.8	310.2	79.7%	1.6%	44	45	89	84.3%	−0.4%
V13	ULNA	Max. length	104	78	182	258.2	228.3	243.2	85.7%	4.2%	35	25	60	91.7%	−7.4%
V14	ULNA	Physiological length	113	95	208	227.4	202.5	214.9	83.2%	3.9%	40	36	76	85.5%	4.2%
V15	ULNA	Min. circumference	112	109	221	37.8	32.0	34.9	80.1%	2.4%	36	38	74	74.3%	−14.9%
V16	ULNA	Max. diameter MS	134	125	259	16.9	14.0	15.5	84.2%	−5.9%	45	45	90	84.4%	−4.4%
V17	ULNA	Min. diameter MS	135	127	262	12.3	9.8	11.1	90.5%	2.9%	45	45	90	85.6%	−15.6%
V18	ULNA	Trochlear notch breadth	140	121	261	21.1	17.7	19.4	84.7%	−2.4%	46	47	93	90.3%	−6.7%
V19	RADIUS	Max. length	120	98	218	239.7	212.5	226.1	85.8%	−1.7%	40	36	76	89.5%	−4.2%
V20	RADIUS	Sag. diameter MS	135	122	257	12.0	9.9	11.0	88.7%	−4.3%	46	48	94	84.0%	−7.1%
V21	RADIUS	Trans. diameter MS	134	126	260	15.6	13.5	14.6	78.1%	−2.5%	45	47	92	70.7%	5.2%
V22	RADIUS	Max. head diameter	115	95	210	23.3	19.8	21.5	90.0%	−2.9%	37	36	73	91.8%	−5.3%
V23	SCAPHOID	Max. length	43	41	84	28.1	24.5	26.3	78.6%	−8.5%	10	19	29	93.1%	−4.7%
V24	SCAPHOID	Max. width	44	40	84	16.7	14.6	15.7	77.4%	−5.0%	10	19	29	86.2%	−24.7%
V25	LUNATE	Length	38	30	66	18.5	15.7	17.1	81.8%	−6.8%	8	12	20	80.0%	−29.2%
V26	LUNATE	Width	38	30	66	18.6	16.0	17.3	87.9%	−8.6%	8	12	20	95.0%	−12.5%
V27	TRIQUETRAL	Max width	20	21	41	15.5	13.9	14.7	82.9%	−5.7%	4	7	11	81.8%	−50.0%
V28	TRIQUETRAL	Max height	19	21	40	16.9	14.6	15.8	85.0%	−11.5%	4	7	11	100.0%	0.0%
V29	PISIFORM	Max. length	10	8	18	14.8	13.1	14.0	72.2%	17.5%	2	5	7	71.4%	40.0%
V30	PISIFORM	Max. width	9	8	17	10.0	9.1	9.6	70.6%	15.3%	2	5	7	85.7%	20.0%
V31	TRAPEZIUM	Max. length	31	25	56	24.7	21.4	23.0	85.7%	3.1%	6	8	14	78.6%	−20.8%
V32	TRAPEZIUM	Height	32	24	56	18.3	16.6	17.4	71.4%	1.0%	5	8	13	84.6%	−7.5%
V33	TRAPEZOID	Length of palmar surf.	34	31	65	17.5	15.3	16.4	76.9%	11.4%	5	11	16	75.0%	−21.8%
V34	TRAPEZOID	Width of dorsal surf.	35	31	66	11.7	10.6	11.1	57.6%	−0.9%	5	11	16	62.5%	25.5%
V35	CAPITATE	Height	55	45	100	24.1	21.3	22.7	82.0%	3.6%	10	20	30	86.7%	−25.0%
V36	CAPITATE	Width of distal base	55	45	100	14.2	12.1	13.2	76.0%	−3.2%	10	18	28	75.0%	7.8%
V37	HAMATE	Max. height	33	36	69	24.5	20.8	22.6	89.9%	2.0%	6	13	19	89.5%	15.4%
V38	HAMATE	Max. width	35	42	77	21.8	18.7	20.3	88.3%	−4.8%	7	14	21	90.5%	−28.6%
V39	MC1	Max. length	64	61	125	46.5	42.0	44.3	82.4%	4.0%	14	20	34	76.5%	−8.6%
V40	MC2	Max. length	91	85	176	69.2	64.1	66.6	75.6%	7.4%	24	29	53	81.1%	−3.6%
V41	MC3	Max. length	86	83	169	68.2	62.7	65.5	72.2%	−0.2%	21	28	49	77.6%	6.0%
V42	MC4	Max. length	67	68	135	58.3	53.6	55.9	74.1%	4.1%	19	25	44	77.3%	−15.6%
V43	MC5	Max. length	60	58	118	54.3	49.8	52.1	78.0%	0.7%	12	20	32	87.5%	−6.7%
V44	STERNUM	Manubrium length	73	67	140	50.5	46.8	48.6	67.9%	−15.8%	20	26	46	78.3%	−5.8%
V45	STERNUM	Body length	66	59	125	101.6	83.9	92.8	80.0%	3.9%	24	23	47	91.5%	8.9%
V46	STERNUM	Total length	45	45	90	150.5	128.2	139.4	82.2%	0.0%	13	16	29	86.2%	−2.9%
V47	STERNUM	Manubrium max. width	61	58	119	58.1	51.6	54.8	72.3%	−7.0%	14	26	40	65.0%	9.9%
V48	STERNUM	Sup. body width	87	63	150	27.1	24.2	25.6	62.0%	−10.8%	28	27	55	70.9%	8.3%
V49	STERNUM	Inf. Body width	73	58	131	34.6	29.3	32.0	74.0%	2.9%	24	24	48	62.5%	−8.3%
V50	1^ST^ RIB	Max. chord	78	71	149	86.1	80.9	83.5	64.4%	−8.8%	23	21	44	72.7%	−15.7%
V51	1^ST^ RIB	Min. chord	82	73	155	55.7	53.5	54.6	56.1%	10.3%	25	22	47	55.3%	1.5%
V52	4^TH^ RIB	Width	37	31	68	17.2	13.4	15.3	94.1%	−4.9%	17	17	34	91.2%	−5.9%
V53	ATLAS	Sagittal diameter	90	93	183	47.1	43.0	45.0	74.3%	−4.1%	37	28	65	76.9%	3.4%
V54	ATLAS	Transverse diameter	63	51	114	81.4	73.3	77.4	81.6%	−1.4%	25	19	44	81.8%	−13.5%
V55	C2	Max. sagittal length	67	50	117	52.0	47.0	49.5	81.2%	9.1%	23	16	39	84.6%	−15.5%
V56	C2	Max. height	95	88	183	40.3	36.6	38.5	77.0%	3.9%	30	23	53	75.5%	−20.3%
V57	C2	Max. breadth sup. facets	100	87	187	47.4	43.8	45.6	75.4%	5.6%	29	27	56	73.2%	−1.7%
V58	C7	Ant. body height	80	73	153	14.1	12.5	13.3	74.5%	−4.2%	27	24	51	80.4%	18.1%
V59	C7	Sag. length	71	46	117	61.9	54.2	58.0	83.8%	−1.7%	22	14	36	80.6%	3.2%
V60	C7	Max width	20	13	33	74.3	66.3	70.3	78.8%	−9.6%	3	7	10	100.0%	0.0%
V61	T1	Ant. body height	80	74	154	16.0	14.1	15.0	74.7%	5.9%	27	29	56	75.0%	26.8%
V62	T1	Sag. length	65	44	109	63.7	56.6	60.2	84.4%	−3.3%	26	16	42	78.6%	15.9%
V63	T1	Width at costal head facets	84	77	161	34.2	30.9	32.6	71.4%	−10.0%	33	30	63	81.0%	1.8%
V64	T12	Ant. body height	99	70	169	24.0	22.5	23.3	65.7%	−4.9%	30	30	60	63.3%	0.0%
V65	T12	Sag. length	53	34	87	76.0	68.0	72.0	79.3%	9.5%	20	11	31	80.6%	−15.9%
V66	T12	Width at costal head facets	104	72	176	45.5	41.0	43.2	72.7%	0.9%	29	27	56	80.4%	−9.3%
V67	L1	Ant. body height	91	76	167	25.8	24.3	25.1	67.7%	1.0%	31	27	58	58.6%	−15.1%
V68	L1	Sag. length	52	43	95	80.0	72.5	76.2	77.9%	2.1%	17	12	29	79.3%	−6.9%
V69	L1	Max. endplate width	89	77	166	48.0	43.2	45.6	75.9%	−3.8%	31	26	57	77.2%	−20.7%
V70	L5	Ant. body height	96	66	162	28.5	26.8	27.7	66.7%	−2.6%	28	26	54	63.0%	10.2%
V71	L5	Sag. length	51	42	93	77.8	71.2	74.5	75.3%	2.7%	13	13	26	73.1%	7.7%
V72	L5	Max. endplate width	92	62	154	52.9	47.3	50.1	79.2%	−15.9%	28	28	56	85.7%	−7.1%
V73	OS COXAE	Max. heigth	111	79	190	215.6	198.6	207.1	78.9%	−10.0%	29	33	62	83.9%	4.4%
V74	OS COXAE	Min. ischium length	115	82	197	57.1	51.1	54.1	79.2%	−12.7%	34	31	65	80.0%	17.3%
V75	OS COXAE	Iliac breadth	69	55	124	158.8	152.5	155.6	65.3%	−0.2%	23	25	48	66.7%	22.3%
V76	OS COXAE	Min. pubis length	63	43	106	70.9	72.5	71.7	NS	-	-	-	-	-	-
V77	OS COXAE	Max. I.P. ramus length	69	47	116	96.8	98.7	97.8	NS	-	-	-	-	-	-
V78	SACRUM	S1 trans. diameter	100	85	185	48.9	43.2	46.1	79.5%	−9.7%	32	34	66	75.8%	4.6%
V79	SACRUM	S1 sagittal diameter	86	72	158	32.6	29.2	30.9	75.9%	6.8%	27	29	56	75.0%	−8.9%
V80	SACRUM	Anterior height	72	37	109	108.7	100.2	104.5	68.8%	10.1%	20	18	38	65.8%	40.6%
V81	SACRUM	Anterior breadth	85	67	152	106.3	106.4	106.4	NS	-	-	-	-	-	-
V82	FEMUR	Epicondylar breadth	118	107	225	83.2	73.0	78.1	92.0%	−2.8%	39	37	76	90.8%	−2.1%
V83	FEMUR	Max. head diameter	135	124	259	48.0	42.0	45.0	86.9%	−2.0%	43	46	89	91.0%	3.9%
V84	FEMUR	Circumference MS	140	128	268	91.3	80.8	86.1	82.1%	0.1%	48	45	93	82.8%	−3.2%
V85	FEMUR	Trans. Diameter MS	143	128	271	27.7	25.0	26.4	72.3%	−0.6%	48	46	94	68.1%	9.9%
V86	FEMUR	Sagittal diameter MS	143	129	272	29.6	26.0	27.8	79.8%	−1.6%	48	48	96	75.0%	−8.3%
V87	FEMUR	Trans. subtroch. diameter	148	134	282	32.0	28.6	30.3	78.0%	−0.7%	47	48	95	72.6%	7.8%
V88	FEMUR	Bicondylar length	142	123	265	446.8	408.6	427.7	79.6%	−6.2%	43	44	87	81.6%	−0.4%
V89	FEMUR	Max. length	143	127	270	449.3	411.9	430.6	80.0%	−5.1%	43	46	89	80.9%	1.0%
V90	FEMUR	Med. cond. max. length	135	110	245	64.4	57.1	60.8	84.9%	0.6%	43	42	85	89.4%	7.3%
V91	FEMUR	Lat. cond. max. length	122	99	221	64.3	57.9	61.1	83.7%	−2.1%	38	40	78	87.2%	−0.7%
V92	TIBIA	Prox. epiphyseal breadth	106	102	208	77.0	67.0	72.0	91.8%	1.3%	36	33	69	91.3%	6.6%
V93	TIBIA	Dist. epiphyseal breadth	130	113	243	48.9	43.1	46.0	86.8%	1.9%	34	36	70	87.1%	−3.6%
V94	TIBIA	Nut. for. circumference	142	144	286	97.2	83.4	90.3	85.0%	−3.7%	47	46	93	89.2%	−4.1%
V95	TIBIA	Nut. for. trans. diameter	143	144	287	26.2	22.6	24.4	81.9%	−4.3%	47	48	95	81.1%	−8.8%
V96	TIBIA	Nut. for. AP diameter	142	144	286	33.9	28.9	31.4	84.6%	−1.6%	47	46	93	86.0%	6.8%
V97	TIBIA	Length	134	127	261	363.2	330.2	346.7	78.5%	−3.4%	46	42	88	84.1%	−3.1%
V98	FIBULA	Max. diameter MS	137	129	266	14.9	13.2	14.1	70.7%	−4.3%	45	43	88	59.1%	−7.2%
V99	FIBULA	Max. length	99	75	174	361.1	332.9	347.0	77.6%	0.4%	28	23	51	86.3%	−1.2%
V100	CALCANEUS	Max. length	114	99	213	82.4	74.8	78.6	79.3%	−0.9%	30	33	63	79.4%	7.6%
V101	CALCANEUS	Middle breadth	115	112	227	43.0	38.4	40.7	82.8%	−0.4%	31	37	68	89.7%	13.0%
V102	TALUS	Length	121	124	245	60.4	53.6	57.0	85.3%	−0.4%	32	41	73	91.8%	3.5%
V103	TALUS	Breadth	119	111	230	42.9	37.8	40.3	83.9%	−1.5%	31	39	70	91.4%	9.6%
V104	CUBOID	Length	101	115	216	38.1	34.0	36.1	75.0%	−1.4%	26	37	63	84.1%	7.4%
V105	CUBOID	Breadth	86	95	181	28.8	25.6	27.2	80.1%	4.7%	19	29	48	77.1%	−5.6%
V106	NAVICULAR	Length	102	122	224	21.3	18.8	20.0	77.7%	−0.4%	30	37	67	77.6%	10.4%
V107	NAVICULAR	Breadth	97	108	205	40.6	36.6	38.6	77.6%	1.5%	29	28	57	80.7%	11.2%
V108	MED CUNEIFORM	Length	103	119	222	26.9	24.4	25.7	80.2%	0.8%	27	37	64	76.6%	−4.3%
V109	MED CUNEIFORM	Height	102	112	214	33.6	30.5	32.1	78.5%	1.7%	27	34	61	83.6%	2.8%
V110	INT CUNEIFORM	Length	91	113	204	19.3	17.6	18.5	76.0%	−2.3%	21	36	57	82.5%	−2.4%
V111	INT CUNEIFORM	Height	85	94	179	22.5	20.1	21.3	77.1%	−1.2%	18	29	47	91.5%	4.8%
V112	LAT CUNEIFORM	Length	90	115	205	25.3	23.0	24.1	76.1%	7.0%	23	40	63	76.2%	10.1%
V113	LAT CUNEIFORM	Height	78	99	177	24.0	21.4	22.7	75.1%	−3.7%	19	31	50	76.0%	21.7%
V114	MT1	Max. length	78	94	172	64.6	59.5	62.0	78.5%	−0.5%	20	27	47	76.6%	5.9%
V115	MT2	Max. length	110	103	213	77.3	71.9	74.6	76.5%	−4.1%	27	32	59	78.0%	−0.3%
V116	MT3	Max. length	104	106	210	72.0	67.0	69.5	74.3%	5.2%	25	31	56	75.0%	−5.4%
V117	MT4	Max. length	100	100	200	70.7	65.6	68.2	75.0%	−2.0%	24	29	53	67.9%	12.9%
V118	MT5	Max. length	94	87	181	71.5	66.2	68.9	73.5%	−0.2%	22	30	52	71.2%	−13.0%
V119	PATELLA	Max. length	68	75	143	43.6	38.0	40.8	85.3%	2.8%	17	24	41	90.2%	−13.5%
V120	PATELLA	Max. breadth	72	75	147	45.6	40.1	42.8	81.6%	0.6%	17	24	41	87.8%	−19.4%
V121	PATELLA	Max. thickness	70	78	148	21.3	18.8	20.0	82.4%	−1.9%	17	26	43	86.0%	−6.1%

### Single variable sex estimation: sectioning points

**Fig. 2:**
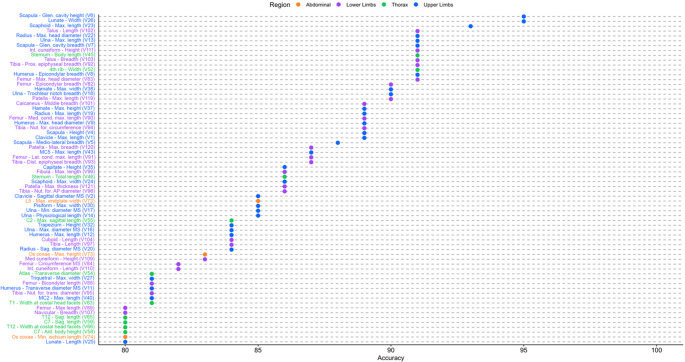
Visualization of sectioning point accuracies (showing only those with an accuracy greater than 80%), arranged from highest to lowest accuracy in the test sample. Measurements are color-coded by anatomical region, as indicated in the legend

Table 2 shows the sectioning points calculated for each measurement, along with their respective accuracy and class discrimination bias. In the validation test, correct classification percentages range from 55.3% (minimum chord of the first rib) to 95.4% (height of the glenoid cavity of the scapula). Two measurements were able to correctly classify all individuals (100%): maximum height of the triquetral and maximum width of C7. However, these two measurements were tested on only 11 and 10 individuals, respectively, a number too small for the result to be considered valid. Excluding measurements with an insufficient sample size, the variable that shows the highest accuracy is the height of the glenoid cavity of the scapula, with an accuracy of 95.4% and a class discrimination bias of −4.7% (Table 2; Fig. 2). In the training sample, the measurement with the highest classification rate was the width of the fourth rib (94.1%; class discrimination bias − 4.9%), although this result decreased slightly in the validation test (91.2%; class discrimination bias − 5.9%). In total, eighteen measurements resulted in correct classifications greater than 90% in the validation test, including measurements from the scapula, long bones of the upper limb, scaphoid, lunate, hamate, sternum, femur, tibia, patella, talus and intermediate cuneiform (Table 2; Fig. 2). Furthermore, a total of 66 measurements, across all post-cranial body regions, reported an accuracy greater than 80% (Fig. 2).

### Multivariable analysis

**Fig. 3:**
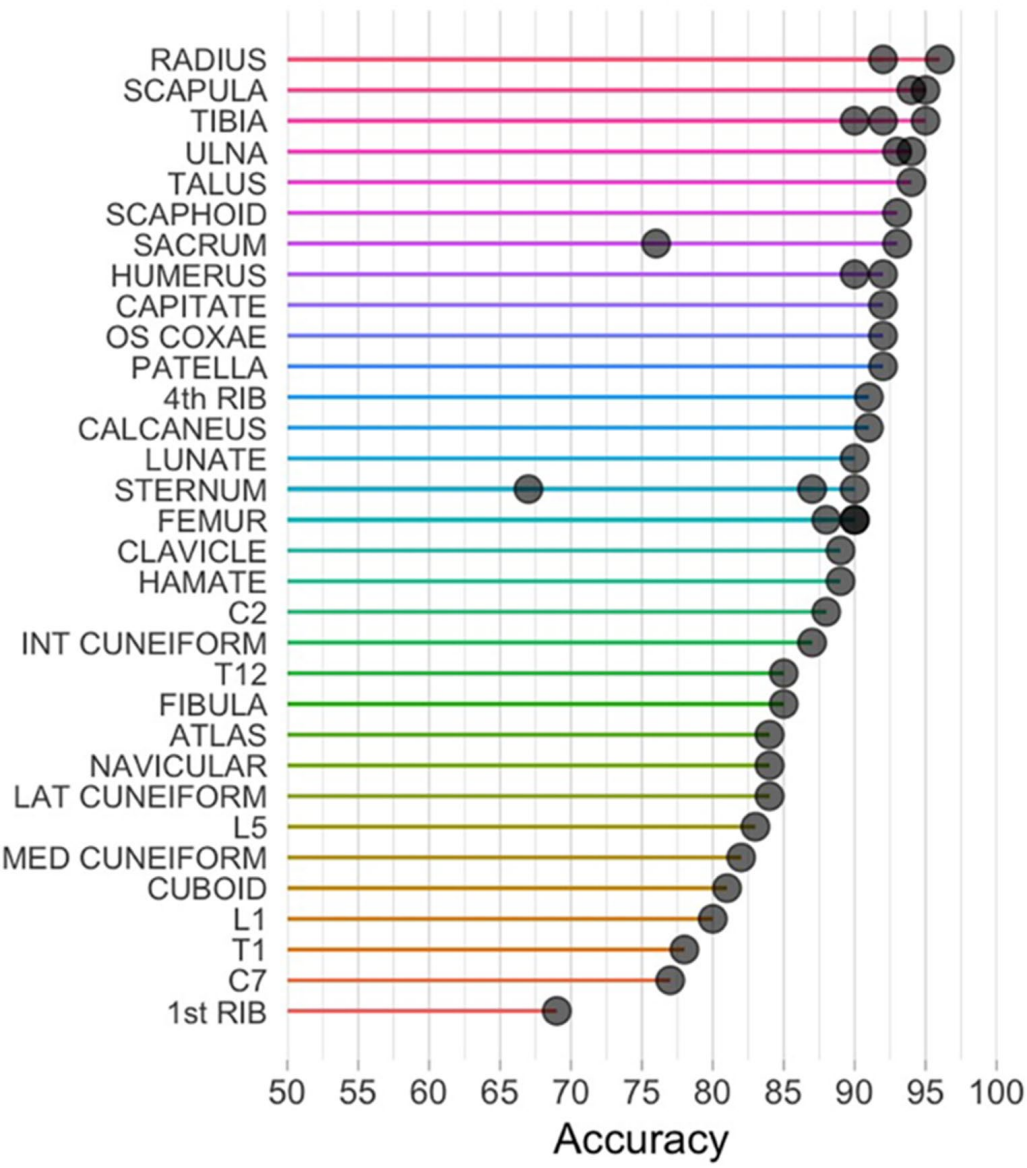
Visualization of the accuracy of multivariable models, with bones ordered by accuracy. Black dots represent the multivariable logistic regression model for each bone

**Fig. 4:**
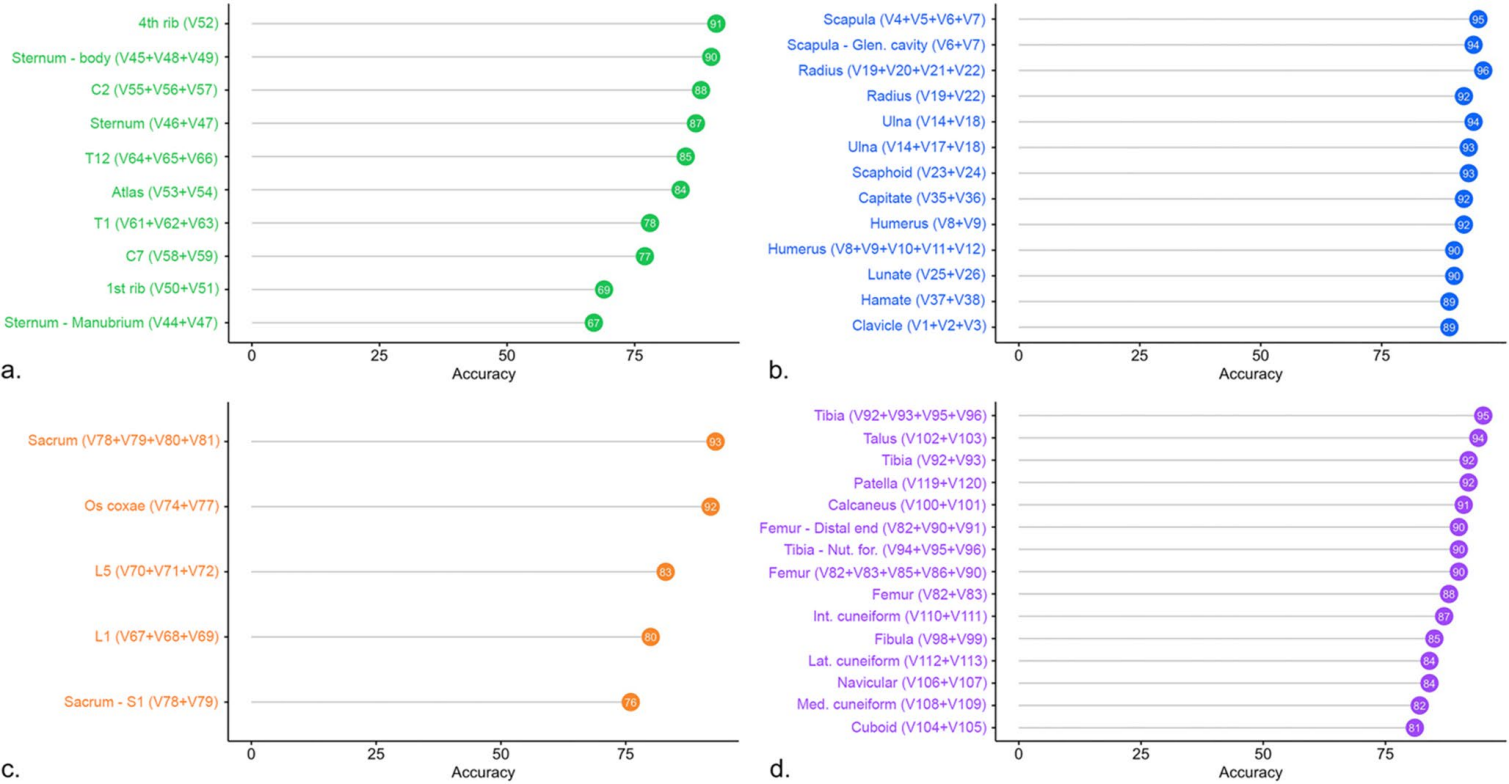
Visualization of the accuracy of multivariable models, divided by anatomical region (**a** = thorax; **b** = upper limb; **c** = abdominal; **d** = lower limb)

Multivariable logistic regression models were developed for each bone. In total, 43 logistic regression models were developed for 32 bones. The models and their corresponding coefficients are reported in Table 3. Table 4 summarizes the results of the models and validity tests. Correct classifications in the test set ranged from 67.6 to 96.8% (Table 4). The manubrium (67.6%; class discrimination bias − 17.7%) and first rib (69.3%; class discrimination bias 15.9%) performed the worst and the radius (96.8%; class discrimination bias 6.0%), scapula (95.3%; class discrimination bias 7.4%), and tibia (95.2%; class discrimination bias − 3.6%) performed the best (Fig. 3). Even with the lower accuracies reported for the manubrium and first rib, total accuracy rates exceeding or approaching 90% were achieved for all body regions (Fig. 4).

**Table 3:** Coefficients for logistic models. To use these algorithms. Multiply each measurement (in millimeters) by its respective coefficient, sum the results, and add the intercept. Predicted probabilities greater than 0.5 are more likely to be males, while values below 0.5 are more likely to be females. Use table 4 for the performance metrics associated with each model

	Bone	Measurements	Intercept
1	CLAVICLE	Max length (0.186) + sagittal diameter MS (0.594) + vertical diameter MS (1.824)	−51.442
2	SCAPULA	Height (0.076) + medio-lateral breadth (0.090) + Glen. cavity height (0.820) + Glen. cavity breadth (0.301)	−57.427
3	SCAPULA (glenoid cavity)	Glen. cavity height (0.968) + glen. cavity breadth (0.432)	−45.931
4	HUMERUS	Epicondylar breadth (0.314) + Max head diameter (0.718)	−49.889
5	HUMERUS	Epicondylar breadth (0.301) + max head diameter (0.678) + sag. diameter MS (−0.092) + trans. diameter MS (0.160) + max length (0.007)	−50.596
6	ULNA	Phys. length (0.098) + min diameter MS (0.858) + trochlear notch breadth (0.682)	−43.602
7	ULNA	Phys. length (0.118) + trochlear notch breadth (0.848)	−41.343
8	RADIUS	Max length (0.139) + sag. diameter MS (3.117) + trans. diameter MS (−0.711) + max head diameter (1.821)	−93.363
9	RADIUS	Max length (0.125) + max head diameter (1.941)	−69.334
10	SCAPHOID	Max length (0.569) + max width (1.072)	−31.479
11	LUNATE	Length (0.803) + Width (1.192)	−33.989
12	CAPITATE	Height (1.226) + width of distal base (0.683)	−36.525
13	HAMATE	Max height (2.000) + max width (0.574)	−56.822
14	STERNUM	Total length (0.151) + manubrium width (0.171)	−30.253
15	STERNUM (manubrium)	Manubrium length (0.055) + manubrium width (0.270)	−17.378
16	STERNUM (body)	Body length (0.146) + sup. body width (0.128) + inf. body length (0.066)	−18.834
17	1 st RIB	Max chord (0.099)	−8.189
18	4th RIB	Width (1.588)	−24.067
19	ATLAS	Sag. diameter (0.347) + trans. diameter (0.344)	−42.066
20	C2	Max sag. length (0.512) + max height (0.050) max breadth sup. facets (0.215)	−36.853
21	C7	Ant. body height (0.366) + sag. length (0.418)	−28.786
22	T1	Ant. body height (0.601) + sag. length (0.308) + width at costal head facets (0.271)	−35.980
23	T12	Ant. body height (0.315) + sag. length (0.205) + width at costal head facets (0.274)	−33.054
24	L1	Ant. body height (0.128) + sag. length (0.134) + max endplate width (0.214)	−23.029
25	L5	Ant. body height (0.447) + sag. length (0.021) + max endplate width (0.547)	−40.818
26	OS COXAE	Min ischium length (1.636) + Max ramus I-P length (−0.640)	−24.826
27	SACRUM	S1 trans. diameter (0.418) + S1 sag. diameter (0.254) + anterior height (0.065) + anterior breadth (−0.187)	−13.053
28	SACRUM (S1)	S1 transverse diameter (0.191) + S1 AP diameter (0.378)	−20.179
29	FEMUR	Epicondylar breadth (0.501) + max head diameter (0.351) + trans. diameter MS (−0.251) + AP diameter MS (0.231) + max length (0.040)	−56.773
30	FEMUR	Epicondylar breadth (0.530) + max head diameter (0.328)	−55.892
31	FEMUR (distal end)	Epicondylar breadth (0.680) + med.cond. max length (0.279) + lat.cond. max length (−0.037)	−67.301
32	TIBIA	Prox epiphyseal breadth (0.522) + Dist epiphyseal breadth (0.373) +for.nut. trans diameter (−0.452) + for.nut. AP diameter (0.480)	−58.446
33	TIBIA	Prox epiphyseal breadth (0.596) + Dist epiphyseal breadth (0.298)	−56.333
34	TIBIA (nutrient foramen)	Nut. for. circum. (0.082) + nut.for. trans diameter (0.195) + nut.for. AP diameter (0.507)	−27.937
35	FIBULA	Max diameter MS (0.218) + max length (0.062)	−24.368
36	CALCANEUS	Max length (0.225) + middle breadth (0.490)	−37.433
37	TALUS	Length (0.404) + breadth (0.422)	−39.916
38	CUBOID	Length (0.283) + breadth (0.647)	−27.944
39	NAVICULAR	Length (0.549) + breadth (0.416)	−27.088
40	MED CUNEIFORM	Length (0.636) + height (0.465)	−31.343
41	INT CUNEIFORM	Length (0.483) + height (0.662)	−23.110
42	LAT CUNEIFORM	Length (0.669) + height (0.560)	−29.067
43	PATELLA	Max length (0.419) + max breadth (0.432)	−35.530

**Table 4:** Accuracies (%t = overall, %M = male correct classification, %F = female correct classification) and class discrimination bias (CD bias) of the multivariable logistic regression models. Refer back to table 2 for variables and to table 3 for respective logistic regression models

	Bone	Measurements	Training sample					Test sample				
			*n*	%T	%M	%F	CD bias	*n*	%T	%M	%F	CD bias
1	CLAVICLE	V1 + V2 + V3	211	93.8%	93.6%	94.1%	−0.5%	64	89.1%	87.5%	90.6%	−3.1%
2	SCAPULA	V4 + V5 + V6 + V7	122	94.3%	93.3%	94.8%	−1.5%	42	95.3%	100.0%	92.6%	7.4%
3	SCAPULA (glenoid cavity)	V6 + V7	245	91.0%	92.0%	90.2%	1.8%	83	94.0%	93.0%	95.0%	−2.0%
4	HUMERUS	V8 + V9	209	91.9%	89.8%	93.4%	−3.6%	77	92.2%	94.6%	90.0%	4.6%
5	HUMERUS	V8 + V9 + V10 + V11 + V12	206	92.2%	90.9%	93.2%	−2.3%	77	90.9%	91.9%	90.0%	1.9%
6	ULNA	V14 + V17 + V18	194	91.2%	89.5%	92.6%	−3.1%	74	93.2%	97.1%	90.0%	7.1%
7	ULNA	V14 + V18	198	88.4%	87.2%	89.3%	−2.1%	75	94.7%	97.1%	92.5%	4.6%
8	RADIUS	V19 + V20 + V21 + V22	175	96.6%	96.0%	97.0%	−1.0%	62	96.8%	100.0%	94.0%	6.0%
9	RADIUS	V19 + V22	184	93.5%	92.5%	94.2%	−1.7%	64	92.2%	90.0%	94.1%	−4.1%
10	SCAPHOID	V23 + V24	83	85.6%	87.5%	83.7%	3.8%	29	93.1%	94.7%	90.0%	4.7%
11	LUNATE	V25 + V26	66	87.9%	85.7%	89.5%	−3.8%	20	90.0%	100.0%	75.0%	25.0%
12	CAPITATE	V35 + V36	100	88.0%	86.7%	89.1%	−2.4%	28	92.9%	94.4%	90.0%	4.4%
13	HAMATE	V37 + V38	69	91.3%	91.7%	90.9%	0.8%	20	89.5%	84.6%	100.0%	−15.4%
14	STERNUM	V46 + V47	75	88.0%	86.8%	89.2%	−2.4%	24	87.5%	76.9%	100.0%	−23.1%
15	STERNUM (manubrium)	V44 + V47	116	70.7%	71.9%	69.5%	2.4%	37	67.6%	60.9%	78.6%	−17.7%
16	STERNUM (body)	V45 + V48 + V49	108	80.6%	78.0%	82.8%	−4.8%	44	90.9%	95.2%	87.0%	8.2%
17	1 st RIB	V50 + V51	138	63.0%	62.1%	63.9%	−1.8%	39	69.3%	77.8%	61.9%	15.9%
18	4th RIB	V52	68	94.1%	96.8%	91.9%	4.9%	34	91.2%	94.1%	88.2%	5.9%
19	ATLAS	V53 + V54	110	85.5%	82.0%	88.3%	−6.3%	44	84.1%	84.2%	84.0%	0.2%
20	C2	V55 + V56 + V57	109	78.9%	71.1%	84.4%	−13.3%	35	88.6%	92.9%	85.7%	7.2%
21	C7	V58 + V59	110	84.6%	80.4%	87.5%	−7.1%	35	77.1%	64.3%	85.7%	−21.4%
22	T1	V61 + V62 + V63	107	86.0%	84.1%	87.3%	−3.2%	37	78.4%	57.1%	91.3%	−34.2%
23	T12	V64 + V65 + V66	75	78.7%	69.2%	83.7%	−14.5%	27	85.2%	90.0%	82.4%	7.6%
24	L1	V67 + V68 + V69	80	78.8%	75.7%	81.4%	−5.7%	26	80.8%	90.0%	75.0%	15.0%
25	L5	V70 + V71 + V72	90	84.4%	82.5%	86.0%	−3.5%	34	83.3%	83.3%	83.3%	0.0%
26	OS COXAE	V74 + V77	110	95.5%	92.7%	97.1%	−4.4%	27	92.6%	83.3%	100.0%	−16.7%
27	SACRUM	V78 + V79 + V80 + V81	81	86.4%	70.8%	93.0%	−22.2%	31	93.6%	86.7%	100.0%	−13.3%
28	SACRUM (S1)	V78 + V79	154	80.5%	75.4%	84.7%	−9.3%	56	76.8%	69.0%	85.2%	−16.2%
29	FEMUR	V82 + V83 + V85 + V86 + V90	186	91.9%	90.1%	93.3%	−3.2%	65	90.8%	89.7%	91.7%	−2.0%
30	FEMUR	V82 + V83	213	91.6%	91.9%	92.0%	−0.1%	70	88.6%	87.9%	89.2%	−1.3%
31	FEMUR (distal end)	V82 + V90 + V91	188	92.6%	91.6%	93.3%	−1.7%	71	90.2%	88.6%	91.7%	−3.1%
32	TIBIA	V92 + V93 + V95 + V96	188	93.1%	93.3%	92.9%	0.4%	62	95.2%	93.3%	96.9%	−3.6%
33	TIBIA	V92 + V93	192	92.2%	92.3%	92.1%	0.2%	64	92.2%	87.5%	96.9%	−9.4%
34	TIBIA (nutrient foramen)	V94 + V95 + V96	286	86.0%	86.1%	85.9%	0.2%	93	90.3%	91.3%	89.4%	1.9%
35	FIBULA	V98 + V99	172	76.7%	71.6%	80.6%	−9.0%	49	85.7%	77.3%	92.6%	−15.3%
36	CALCANEUS	V100 + V101	209	83.3%	79.2%	86.7%	−7.5%	61	91.8%	84.4%	100.0%	−15.6%
37	TALUS	V102 + V103	230	87.8%	86.5%	89.1%	−2.6%	70	94.3%	89.7%	100.0%	−10.3%
38	CUBOID	V104 + V105	181	79.0%	80.0%	77.9%	2.1%	48	81.3%	82.8%	79.0%	3.8%
39	NAVICULAR	V106 + V107	204	77.6%	79.4%	75.3%	4.1%	57	84.2%	75.0%	93.1%	−18.1%
40	MED CUNEIFORM	V108 + V109	214	80.8%	82.1%	79.4%	2.7%	61	82.0%	79.4%	85.2%	−5.8%
41	INT CUNEIFORM	V110 + V111	179	80.5%	81.9%	78.8%	3.1%	47	87.2%	89.7%	83.3%	6.4%
42	LAT CUNEIFORM	V112 + V113	176	81.8%	84.9%	77.9%	7.0%	50	84.0%	80.7%	89.5%	−8.8%
43	PATELLA	V119 + V120	141	85.8%	89.0%	82.4%	6.6%	41	92.3%	95.8%	88.2%	7.6%

*n* number of individuals, *%T* percentage of accuracy in the total sample, %*M* percentage of accuracy in the male sample, %*F* percentage of accuracy in the female sample

A logistic regression model was also developed for the fourth rib, even though the study considered only a single metric variable for this bone (the width of the sternal end). The logistic regression results confirm the utility found with the application of the sectioning point and provides the posterior probability. In contrast, no logistic regression models were developed for the metacarpals and metatarsals, as only a single measurement was examined for these bones, and the resulting accuracies rates from the sectioning points were insufficient to investigate further with logistic regression analysis.

Overall, the logistic regression models reported class discrimination bias values within acceptable thresholds in both the training and test samples (Table 4). Notably, the models for the radius and scapula, which demonstrated the highest classification accuracies, exhibited class discrimination bias values slightly above the recommended threshold in the test sample (6.0% and 7.4%, respectively). However, these deviations were minimal, and the corresponding models in the training sample remained within acceptable limits. There are exceptions in which models exhibited high class discrimination bias in the test sample, despite having balanced and acceptable class discrimination bias values in the training sample. These cases generally correspond to models developed with a smaller number of individuals, such as those for certain carpal bones (lunate and hamate) and vertebrae (C2, C7, T1, T12, L1).

## Discussion

This study developed an easily applied and statistically substantiated method for biological sex estimation specific to the Italian population, employing both simple and multivariable metric analyses of postcranial bones. Total correct classifications in both types of models but especially in the multivariable models, were greater than 90% indicating its efficacy. Because of this high performance across the entire body, the metric analysis of postcranial elements is considered second only to the evaluation of the morphological features of the pelvis, while demonstrating better validity than both metric and non-metric features of the skull [2].

### Intra- and inter- observer agreement

One of the aspects that makes the metric approach appealing is its objectivity and repeatability [43, 44]. However, defining and identifying the anatomical landmarks required for the measurements is not always straightforward, leading to a potential limitation in the reliability of the measurements. Demonstrating the complexity of identifying anatomical landmarks, some measurements of this study showed rTEM values exceeding the standard acceptability thresholds for intraobserver (rTEM > 1.5%) and interobserver (rTEM > 2.0%) error. Most of these measurements relate to the diameters at the midshaft of long bones, which is unsurprising based on previous literature that highlights these measurements as the most susceptible to measurement errors [37]. Consequently, a recent study by Langley and colleagues (2018) [37], focused on the quantification of osteometric error, suggested replacing the traditional measurement of diameters, which depend on position (i.e., transverse diameter, sagittal diameter), with minimum and maximum diameters, as these showed lower rTEM values. The present study still considered the traditional measurement of diameters, except for the ulna. However, the results of this study revealed a significant exception to the conclusions of Langley and colleagues (2018) [37]. Contrary to their prediction, the minimum and maximum diameters of the ulna, used in accordance with their recommendations, exhibited higher rTEM values than the traditional transverse and anteroposterior diameters considered for other long bones of the limbs. Another possibility is that smaller measurements may yield greater negative outcomes in TEM values. However, measurements of the carpal bones, despite their small size, did not exhibit rTEM values beyond the thresholds of acceptability, highlighting the high reliability of these measurements. This result suggests that the error associated with the measurements of the diameters may be intrinsic and not influenced by the methodological approach used, highlighting the need for further research to fully understand the causes of this discrepancy and to improve the precision of osteometric measurements.

Despite some measurements exhibiting rTEM values above the stricter thresholds adopted in this study (< 1.5% for intra-observer and < 2.0% for inter-observer error), all values remained below the broader acceptability limit of < 5% reported in the literature. Furthermore, the calculation of the reliability coefficient indicates that all measurements meet the standard threshold value (*R* > 0.95) [40], indicating strong agreement between measurement repetitions. The differential findings between the repeatability methods highlight how different methodological approaches can lead to different conclusions.

### Differences between sexes

The Italian sample showed strong sexual dimorphism in size, demonstrated by the majority of measurements exhibiting significant differences between males and females, which is also reflected in the accuracies achieved by the sectioning points (Table 2). The results of the current study (Table 2) match or exceed those reported in the literature for other populations [2, 5, 6, 45, 46]. Contrary to most measurements, minimum pubic length, maximum ischiopubic ramus length, and anterior width of the sacrum showed greater dimensions in females. This was expected due to the adaptation of the female pelvis for childbirth, which results in longer pubic lengths and a more lateral growth of the ischiopubic ramus [47, 48]. Consistent with previous research, joint measurements are the best indicators for sex estimation based on their high accuracies, while measurements of the maximum length of long bones and midshaft diameters have less utility based on their lower accuracies, although they still provide good classification rates [2, 7, 27]. Indeed, the literature highlights that sexual dimorphism is more pronounced for body weight than for stature, with a sexual dimorphism approximately of 18% for body mass, while only 8% for stature [49]. Joint dimensions are therefore particularly dimorphic, as these areas are correlated with body weight load and muscle attachment, with males tending to have larger and more robust joints to support greater muscle mass and physical strength [50].

### Single variable and multivariable analyses

Given the high level of sexual dimorphism of the Italian sample and the ability for metric data to collect precise information, the sectioning point results were excellent in terms of accuracy. This extremely simple approach, was capable of achieving accuracies over 80% for all body regions, and even 90% for some skeletal elements, such as the scapula, long bones of the upper limb, femur, tibia, sternum, fourth rib, talus, scaphoid and lunate. The application of sectioning points has the advantage of being computationally simple, quick, and can be used in a variety of biological anthropology contexts because it only requires a single variable. Therefore, this approach can also be used on fragmentary skeletal elements. The results of the multivariable analysis generally showed an improvement in accuracy compared to single variable analysis, which is also expected based on previous literature [e.g. 2, 51–55]. For each logistic regression model, the correct classification rate obtained from the test sample reported, in all body regions, was close to or exceeded 90%. The fact that the entire post-cranial skeleton exhibits comparable levels of sexual dimorphism when considered through a multivariable lens is truly remarkable. Unlike morphological methods, which are typically limited to specific sexually dimorphic traits in the pelvis and skull, the metric approach offers the advantage of providing accurate sex estimations across all post-cranial regions. This highlights a key strength of the metric approach in direct contrast to the most popular morphological approaches for sex estimation: its applicability in various conservation contexts, including commingled or fragmentary remains, regardless of the number or type of elements preserved.

### Performance based on skeletal elements and variables

The scapula emerged as one of the most sexually dimorphic bones in the Italian population, with classification rates exceeding 95% in both single variable analysis and multivariable models. The glenoid cavity was the variable achieving the highest accuracy (95.4%, class discrimination bias − 4.7%) across all measurements in this study. This contrasts with findings from other populations, where maximum scapular height was often the most accurate measurement [e.g. 8, 56–59]. The glenoid cavity also showed better resistance to taphonomic changes, while the scapular body was more prone to postmortem fractures, making it a suitable area. This study’s results for multivariable scapula model (95.3%) surpass previous findings for the Italian population of 92.6% [60] and 95% accuracy [32]. Multivariable analysis of the scapula proved to be an accurate method for sex estimation in various populations, with several studies reporting accuracies over 90% [2, 8, 58, 60–64]. Similarly, Spradley et al. (2015) [6] found 95.6% accuracy in a Hispanic sample, and Moore et al. (2016) [5] reported 93.5% accuracy in a Colombian sample, confirming the consistency of the scapula’s predictive ability for sex estimation.

The long bones of the upper limb have also proven to be particularly accurate for sex estimation using metric approaches. The radius achieved the highest accuracy levels in multivariable analysis, reporting a validity of 96.8% (class discrimination bias 6%) for the equation that combines all four analyzed variables. The results align with findings from other population contexts, with accuracies consistently over 90% [2, 5, 6, 9, 12, 65–68]. Similarly to our study, Spradley and Jantz (2011) [2] found that the radius was the skeletal element providing the highest accuracy in multivariable analysis in an African American sample. In contrast, the radius had a slightly lower accuracy of 85.6% for the White American sample [2]. Likewise, in similar studies, the accuracy of multivariable analysis for the radius was around 90% for Colombian [5] and Hispanic [6] samples. However, these cited studies did not consider the metric analysis of the radial head. In the current study, the radial head measurements was the variable with the most utility in a single variable approach (91.8%) and the most significant in the multivariable logistic regression models. Previously published findings using an American sample also demonstrated the radial head could achieve high accuracies (94%) [69], as well as in a Portuguese sample (90.4%) [70] and a Thai sample (92%) [71]. Regarding the humerus, the best metric variable found was the epicondylar width, which showed better validity than the humeral head in terms of classification rate (91.2% vs. 89.3%, respectively) and class discrimination bias (−1.4% vs. −10.4%, respectively). This result contrasts with previous reports for the CAL cemetery collection, where a similar result was reported for the diameter of the humeral head, but a much lower accuracy was achieved (81.6%) for the epicondylar width [7]. The current study had a much larger sample (400 individuals vs. 164 individuals) compared to Selliah and colleagues (2020) [7], allowing for greater variability. Additionally, the comparable performance in the training and testing sets in the current study validate its performance. The ulna represented an exception among long bones, as it is the only one reporting better validity in single variable analysis for maximum length (91.7%), although it shows a class discrimination bias slightly over the recommended standards (−7.4%). Our results align with other studies that identify the maximum length of the ulna as the most accurate measurement for this bone across different populations [2, 5, 6]. Additionally, the current study introduced the measurement of the trochlear notch breadth, a rarely investigated area in osteometric studies. The research by Zapico and Adserias-Garriga (2021) [72] indicated this area as extremely valid, with an accuracy of 91.3% for the minimum olecranon breadth (similar to the trochlear notch breadth though not identical) in a small sample of European Americans. The current study highlights the validity of this new measurement, showing it can correctly discriminate between sexes in 90.3% of cases and is a significant variable in the ulna’s multivariable analysis.

The long bones of the lower limb, particularly the femur and tibia, have also proven to be particularly accurate in our study. In the multivariable analysis, the bone models exceed 90% accuracy, and reach 95.2% (class discrimination bias − 3.6%) for the tibia model, which combines epiphyseal widths and diameters measured at the nutrient foramen. The femur and tibia have been extensively studied using metric approaches, reporting high accuracies across various population contexts [e.g. 2, 5, 6, 11, 73–77]. A particularly dimorphic skeletal portion is related to the knee joint. The femoral epicondylar width and the proximal tibial , epiphyseal width achieved accuracies over 90%, with class discrimination bias contained within recommended thresholds, in single variable analysis. These measurements are reported to be the most suitable in White American and Black American populations [2], and have also shown accuracies greater than 90% in various European populations [e.g., 11, 78, 79]. The strong sexual dimorphism in this skeletal area is attributed to the knee region’s correlation with body weight load and muscle attachment [50]. Consequently, the patella also allows for effective sex estimation. In this study, single variable analysis of three metric dimensions of the patella yielded an accuracy of up to 91%, improving to 92.3% with multivariable analysis, confirming its validity for sex estimation through metric approaches [80–86].

In contrast, some skeletal elements revealed lower validity for metric sex estimation. The fibula emerged as the least sexually dimorphic long bone, as previously indicated by the literature [2, 5, 6, 39]. The first rib showed low accuracies (below 70%) with both sectioning points and multivariable analyses. This result contrasts with a Polish study that reported an accuracy of up to 90% [87]. However, the original study evaluated nine metric characteristics, while the present study considers only two. The vertebral column was the only body region where accuracy did not exceed 90%, although the multivariable analysis improved accuracy for all considered vertebrae, with validity ranging from 77.1% for C7 to 88.6% for C2. However, some studies indicate that surpassing 90% accuracy is possible for different vertebrae in various populations, often utilizing more metric variables than those selected for this study [14, 15, 88–92]. Pelvic measurements were not particularly useful for sex estimation with single variables, with the highest classification rate for the height of the os coxae at 83.9% and no sacral measurement exceeding 75%, consistent with the literature [93–95]. Even if multivariable models considerably improved the accuracy for these bones, achieving 92% accuracy for the os coxae and 93% for the sacrum, they still exhibited class discrimination bias beyond the threshold recommended for forensic applications, which could potentially compromise the validity of the estimations. The maximum length of the metacarpals and metatarsals demonstrated limited sexual dimorphism in the Italian population, with the highest accuracy of 87.5% for the fifth metacarpal and 78.2% for the second metatarsal. These results are consistent with previous studies indicating that single-variable length measurements of these bones rarely exceed 80% accuracy [96–103].

Other skeletal elements have proven valid for sex estimation but are limited in applicability due to greater susceptibility to taphonomic alterations, thus resulting in smaller sample sizes for our study. This is particularly evident for the carpal bones, for which the sample size was extremely limited due to the difficulty of recovering these small bones in this cemetery burial context [34]. The osteometric study of carpals for sex estimation has only been considered since 2008, with the pioneering research by Sulzmann et al. (2008) [20]. The current study further supports the potential of these small hand bones, reaching accuracies of up to 93%, in line with findings from other populations [20, 22, 104, 105]. Another example is the thoracic area, with the sternum and the fourth rib. The width at the sternal end of the fourth rib has also shown high classification rates in other populations [16, 19, 106, 107]. It is known that the thoracic cavity volume is about 10% smaller in females than in males of the same stature [108], which can explain the observed sexual dimorphism. Yet, it is particularly susceptible to taphonomic alterations, as the central thoracic ribs (fourth to tenth) are more prone to post-mortem fractures and environmental factors [109, 110], resulting in a limited number of individuals in the sample (68 in the training sample and 34 in the test sample). Additionally, its dimensions appear to be influenced by the individual’s age, showing an increasing trend with age [107, 111]. Given that our sample contains many older individuals, the sectioning point obtained should be applied cautiously, and future validation is necessary in younger individuals.,

### Limitations

Despite the robustness of the methodology and the relatively large sample size, this study has certain limitations that should be acknowledged. The main one is related to the preservation of skeletal remains. Although the overall sample size is substantial, taphonomic alterations have affected the completeness of certain bones, reducing data availability. As a result, some anatomical regions of the skeleton are underrepresented in the analysis, such as the carpal bones and vertebral column, which potentially impacts the accuracy and generalizability of the sex estimation models for these bones. Another limitation concerns the population-specificity of the developed method. While this study focuses on a Northern Italian population, it is important to consider the regional variability that may exist within Italy itself. Factors such as geographical diversity, historical migrations, and genetic variability may affect the generalizability of these results across the entire Italian population. Therefore, while the methodology shows high accuracy for the sample analyzed, and the testing set provides a form of validation, further research is needed to assess whether it can be generalized and applied on a greater scale across Italy.

## Conclusion

The collected data enabled the development and validation of a metric method for sex estimation specific to the Italian population. Whereas some components of the biological profile are not substantially impacted by population variation [e.g., 112, 113] metric data are impacted by population variation. Therefore, the current research provides a notable achievement for forensic anthropology in Italy, as it provides easy to apply yet computationally robust single variable and multivariable models to estimate sex. Up to this point, sex estimation was explored primarily in smaller samples [e.g., 7, 30] and/or only using a limited number of variables or elements [e.g., 29, 32, 33, 60, 114, 115] on Italian individuals, and when in a situation where a model was not possible, there was reliance on standards developed in other countries. Since sectioning points and their classification rates were provided for all standard postcranial measurements, and multivariable analysis was conducted on a bone-by-bone basis, the developed methodology is also applicable in contexts of commingled remains and fragmented skeletal remains.

The results presented in this study highlight the high accuracy of the metric approach for sex estimation and contribute to the growing literature illustrating the advantages of using multiple variables to increase confidence in our estimations. Multivariable logistic regression models displayed accuracies above or close to 90% with a contained class discrimination bias across all skeletal regions. The sectioning points developed allowed for an equally accurate and quick estimation of biological sex, as evidenced by 18 measurements exceeding 90% accuracy in the validation test. The models presented in the current study do not require any software or specialized training, allowing for immediate adoption by forensic laboratories across the country. We believe the large sample size adequately captures the range of human variation, and the testing sample acts as a strong validation set, however we encourage colleagues to test the developed models with additional external samples to ensure generalizability.

## Supplementary Information

Below is the link to the electronic supplementary material.

Supplementary Material 1

## Data Availability

The datasets generated during and/or analysed during the current study are available from the corresponding author [PM] on reasonable request.
