# Supplementary material for: Metric analysis of the postcranial skeleton: a comprehensive approach for biological sex estimation in an Italian population
Source: Int J Legal Med. 2025 Oct 3;140(1):441–61. doi: 10.1007/s00414-025-03599-8 (PMC12808299; doi:10.1007/s00414-025-03599-8)
Supplement: Supplementary file 1 — Supplementary Material 1 [file 414_2025_3599_MOESM1_ESM.docx]

**Supplementary Materials**

**Metric analysis of the postcranial skeleton: a comprehensive approach for biological sex estimation in an Italian population**

Authors: Paolo Morandini, MS^1*^, Lucie Biehler-Gomez, PhD^1*^, Kyra Stull, PhD^2^, Cristina Cattaneo, MD, PhD^1^

(*co-first)

^1^ LABANOF (Laboratorio di Antropologia e Odontologia Forense), Department of Biomedical Science for Health, University of Milan, 20133, Milan, Italy

^2^Department of Anthropology, University of Nevada, Reno, Nevada

**Corresponding author**

Paolo Morandini, MS

LABANOF (Laboratorio di Antropologia e Odontologia Forense)

Department of Biomedical Science for Health

University of Milan

paolo.morandini@studenti.unimi.it

**Table A**. Definitions, measurement procedures and references of the metric variables analyzed in this study.

| Variable | Bones | Metric variable | Definition | Measurement procedure | Reference |
| --- | --- | --- | --- | --- | --- |
| V1 | CLAVICLE | Max. length | The maximum distance between the sternal and acromial ends of the clavicle. | Place the clavicle on the osteometric board, with the sternal end against the vertical end board. Press the movable upright against the acromial end and rotate the bone until the maximum length is obtained. | Langley et al., 2016 [1] |
| V2 | CLAVICLE | Sagittal diameter MS | The antero-posterior distance measured at the midshaft of the clavicle. | Identify the midpoint of the clavicular shaft using the osteometric board and mark it with a pencil. At the marked point, measure the anteroposterior diameter using the caliper. | Moore-Jansen et al., 1994 [2] |
| V3 | CLAVICLE | Vertical diameter MS | The supero-inferior distance measured at the midshaft of the clavicle. | Identify the midpoint of the clavicular shaft using the osteometric board and mark it with a pencil. At the marked point, measure the supero-inferior diameter using the caliper. | Moore-Jansen et al., 1994 [2] |
| V4 | SCAPULA | Height | The distance between the superior point of the superior angle and the inferior point of the inferior angle of the scapula. | Place the scapula on the osteometric board, with the superior angle against the vertical end board. Press the sliding pointer against the inferior angle and rotate the bone until the maximum measurement is obtained. | Langley et al., 2016 [1] |
| V5 | SCAPULA | Medio-Lateral Breadth | The distance from the inferior point of the glenoid cavity margin to the inferomedial point of the scapular spine on the vertebral border. | Position the fixed arm of the caliper on the inferior margin of the glenoid cavity. Extend the sliding arm to the vertebral border, moving it until the inferomedial point of the scapular spine is located. | Modified from Langley et al., 2016 [1] |
| V6 | SCAPULA | Glen. cavity height | The distance from the superior point on the margin of the glenoid cavity to the inferior point on the same margin, measured perpendicular to the width of the glenoid cavity. | Place the fixed arm of the caliper on the superior point of the glenoid cavity margin and measure the distance to the corresponding inferior point. | Langley et al., 2016 [1] |
| V7 | SCAPULA | Glen. cavity breadth | The maximum distance between the ventral and dorsal margins of the glenoid cavity, measured perpendicular to the height of the glenoid cavity. | Position the arms of the caliper on the ventral and dorsal margins of the glenoid cavity to obtain the maximum measurement perpendicular to the height of the glenoid cavity. | Langley et al., 2016 [1] |
| V8 | HUMERUS | Epicondylar breadth | The distance from the most prominent point of the lateral condyle to the corresponding projection on the medial epicondyle. | Position one arm of the caliper against the most prominent point of the lateral epicondyle and measure to the corresponding point on the medial epicondyle. | Langley et al., 2016 [1] |
| V9 | HUMERUS | Max. head diameter | The maximum distance between two points located on the margin of the humeral head. | Rotate the caliper along the edge of the articular surface until the maximum diameter is found. Do not include any lipping that may be present on the perimeter of the articular surface. | Langley et al., 2016 [1] |
| V10 | HUMERUS | Sagittal diameter MS | The anteroposterior distance measured at the midshaft of the humerus. | Identify the midpoint of the humeral shaft using the osteometric board and mark it with a pencil. At this marked point, measure the anteroposterior diameter using the caliper. | Moore-Jansen et al., 1994 [2] |
| V11 | HUMERUS | Transverse diameter MS | The distance between the medial and lateral sides measured at the midshaft of the humerus. | Identify the midpoint of the humeral shaft using the osteometric board and mark it with a pencil. At this marked point, measure the transverse diameter using the caliper. | Moore-Jansen et al., 1994 [2] |
| V12 | HUMERUS | Max. length | The distance from the superior point of the humeral head to the inferior point of the humeral trochlea. | Place the humerus on the osteometric board so that its long axis is parallel to the tool. Position the humeral head against the vertical end board. Press the movable upright against the humeral trochlea and rotate the bone until the maximum length is obtained. | Langley et al., 2016 [1] |
| V13 | ULNA | Max. length | The distance from the most proximal point of the olecranon to the most distal point of the styloid process. | Position the ulna on the osteometric board with its proximal end against the vertical end board. Press the movable upright against the distal end while rotating the bone to find the maximum length. | Langley et al., 2016 [1] |
| V14 | ULNA | Physiological length | The distance between the deepest point on the coronoid process's articular surface at the guiding ridge and the lowest point on the distal articular surface of the ulna. | Position the ulna on the osteometric board with the most protruding point of the coronoid process against the vertical end board. Press the movable upright against the distal articular surface, keeping the ulna parallel to the board. Exclude the styloid process or the groove between the styloid process and the distal articular surface. | Langley et al., 2016 [1] |
| V15 | ULNA | Min circumference | The least circumference measured near the distal end of the bone. | Wrap a measuring tape around the diaphysis of the ulna in its distal portion, adjusting the tape until the smallest circumference is found. | Langley et al., 2016 [1] |
| V16 | ULNA | Max diameter MS | The maximum diameter of the ulna measured at midshaft. | Identify the midpoint of the diaphysis using an osteometric board and mark it with a pencil. At the marked point, use the caliper with one hand while rotating the bone with the other to find the maximum diameter. | Langley et al., 2016 [1] |
| V17 | ULNA | Min diameter MS | The minimum diameter of the ulna measured at midshaft. | Identify the midpoint of the diaphysis using an osteometric board and mark it with a pencil. At the marked point, use the caliper with one hand while rotating the bone with the other to find the minimum diameter. | Langley et al., 2016 [1] |
| V18 | ULNA | Trochlear notch breadth | The medio-lateral distance of the olecranon measured at the trochlear notch. | Place the caliper arms on the most medial and lateral points of the trochlear notch. | New measurement |
| V19 | RADIUS | Max. length | The distance from the most proximal point on the radial head to the tip of the styloid process, without considering the bone's long axis. | Position the proximal end against the vertical end board and press the movable upright against the distal end. Adjust the bone until the maximum length is reached. | Langley et al., 2016 [1] |
| V20 | RADIUS | Sagittal diameter MS | The antero-posterior distance measured at the midshaft of the radius. | Identify the midpoint of the radial shaft using an osteometric board and mark it with a pencil. At the marked point, measure the antero-posterior diameter using the caliper. | Moore-Jansen et al., 1994 [2] |
| V21 | RADIUS | Transverse diameter MS | The distance between the medial and lateral sides measured at the midshaft of the radius. | Identify the midpoint of the radial shaft using the osteometric board and mark it with a pencil. At this marked point, measure the transverse diameter using the caliper. | Moore-Jansen et al., 1994 [2] |
| V22 | RADIUS | Max. head diameter | The maximum diameter of the radial head measured along the margin articulating with the ulna | Place the caliper on the articulating margin of the radial head and rotate the bone to find the maximum diameter. | Langley et al., 2016 [1] |
| V23 | SCAPHOID | Max. length | Maximum distance between the tubercle and the articular facet for the lunate. | Position the tubercle against one arm of the caliper and the medial facet articulating with the lunate against the other arm. Rotate the bone until the maximum distance is obtained. | Sulzmann et al., 2008 [3] |
| V24 | SCAPHOID | Max. width | Maximum distance between the protruding points of the scaphoid body. | Place the caliper arms against the most protruding points on both sides of the scaphoid body. Rotate the caliper arms to achieve the maximum measurement. | Sulzmann et al., 2008 [3] |
| V25 | LUNATE | Length | Maximum distance between the medial side of the lunate and the lateral horns. | Place the two "horns" against one arm of the caliper and close the other arm against the most protruded point on the rounded medial side. | Modified from Sulzmann et al., 2008 [3] |
| V26 | LUNATE | Width | Distance between the palmar and dorsal portions of the lunate. | Position the dorsal side of the bone against one arm of the caliper so that it rests on two protruding points. Move the sliding arm of the caliper against the palmar portion of the bone to obtain the maximum measurement. | Modified from Sulzmann et al., 2008 [3] |
| V27 | TRIQUETRAL | Max. Width | Maximum distance between the palmar and dorsal sides of the triquetral. | Place one arm of the caliper against the most protruding point of the articular facet for the lunate and the other arm dorsally at the upper part of the articular facet for the pisiform. Orient the caliper to achieve the maximum measurement. | Sulzmann et al., 2008 [3] |
| V28 | TRIQUETRAL | Max. Height | Maximum distance between the superior and inferior sides of the triquetral. | Place the articular facet for the lunate flat against one arm of the caliper and the most protruding point beyond it against the other arm, avoiding rotation of the caliper. | Sulzmann et al., 2008 [3] |
| V29 | PISIFORM | Max. length | The maximum distance from the distal to the proximal portion of the bone. | Place the dorsal and proximal sides against the arms of the caliper. Adjust the caliper orientation to achieve the maximum measurement. | Sulzmann et al., 2008 [3] |
| V30 | PISIFORM | Max. width | Maximum distance perpendicular to the previous measurement (V29: Maximum Length). | Orient the caliper to achieve the greatest width perpendicular to the previous length measurement (V29). | Sulzmann et al., 2008 [3] |
| V31 | TRAPEZIUM | Max. length | Maximum proximo-distal distance between the projection of the articular facet for the second metacarpal and the palmar crest | Position one arm of the caliper against the most distal point of the articular facet for the second metacarpal and the other arm against the proximal projection of the palmar crest to obtain the maximum measurement. | Sulzmann et al., 2008 [3] |
| V32 | TRAPEZIUM | Height | Maximum distance between the articular facets for the first metacarpal and the pair of facets for the scaphoid and trapezoid. | Hold the trapezium with the articular facet for the first metacarpal positioned superiorly. Place one arm of the caliper on the most superior point of the facet for the first metacarpal and the other arm on the most inferior point where the trapezoid facet meets the scaphoid facet. | Sulzmann et al., 2008 [3] |
| V33 | TRAPEZOID | Length of palmar surf. | Maximum distance measured at the base of the "boot”. | Position one arm of the caliper against the lateral part of the bone so that it rests at two protruding points, and the other arm against the medial extremity's most protruding point at the "toe of the boot". | Sulzmann et al., 2008 [3] |
| V34 | TRAPEZOID | Width of dorsal surf. | Maximum proximo-distal distance measured on the dorsal surface. | Position the caliper arms on the most protruding distal and proximal points on the dorsal surface, perpendicular to V33. | Sulzmann et al., 2008 [3] |
| V35 | CAPITATE | Height | Distance between the distal side and the proximal head. | Place the caliper arms on the most protruding point of the proximal head and on the distal base of the bone, ensuring contact at the two most protruding points. Rotate the caliper arms if necessary to achieve the maximum height while maintaining contact with two points of the base. | Modified from Sulzmann et al., 2008 [3] |
| V36 | CAPITATE | Width of distal base | Distance between the lateral articular facets for the second and third metacarpals. | Place one caliper arm flat against the facet for the third metacarpal and the other arm against the lateral facet for the second metacarpal on opposite sides to achieve the maximum width. Rotation of the caliper is usually unnecessary. | Sulzmann et al., 2008 [3] |
| V37 | HAMATE | Max. height | Maximum dorso-palmar distance measured from the most protruding palmar point of the hamulus to the dorsal base of the hamate. | Place the dorsal base of the hamate against one caliper arm, then place the other arm on the most protruding palmar point of the hamulus. Adjust the positioning of the caliper on the dorsal base if necessary to account for uneven surfaces and obtain the maximum measurement. | Sulzmann et al., 2008 [3] |
| V38 | HAMATE | Max. width | Maximum proximo-distal distance measured from the most protruding palmar point of the hamulus to the most protruding proximal point of the hamate. | Position the most protruding palmar point of the hamulus and the dorsal base of the bone against one caliper arm, and the other arm against the most protruding proximal point. | Sulzmann et al., 2008 [3] |
| V39 | MC1 | Max. length | Maximum distance between the proximal and distal ends of the first metacarpal. | Position one caliper arm against the most protruding point at the proximal end and the other arm against the most protruding point of the distal head. If necessary, rotate the bone on its axis to obtain the maximum measurement. | Scheuer e Elkington, 1993 |
| V40 | MC2 | Max. length | Maximum distance between the proximal and distal ends of the second metacarpal. | Position one caliper arm against the most protruding point at the proximal end and the other arm against the most protruding point of the distal head. If necessary, rotate the bone on its axis to obtain the maximum measurement. | Scheuer e Elkington, 1993 [4] |
| V41 | MC3 | Max. length | Maximum distance between the proximal and distal ends of the third metacarpal. | Position one caliper arm against the most protruding point at the proximal end and the other arm against the most protruding point of the distal head. If necessary, rotate the bone on its axis to obtain the maximum measurement. | Scheuer e Elkington, 1993 [4] |
| V42 | MC4 | Max. length | Maximum distance between the proximal and distal ends of the fourth metacarpal. | Position one caliper arm against the most protruding point at the proximal end and the other arm against the most protruding point of the distal head. If necessary, rotate the bone on its axis to obtain the maximum measurement. | Scheuer e Elkington, 1993 [4] |
| V43 | MC5 | Max. length | Maximum distance between the proximal and distal ends of the fifth metacarpal. | Position one caliper arm against the most protruding point at the proximal end and the other arm against the most protruding point of the distal head. If necessary, rotate the bone on its axis to obtain the maximum measurement. | Scheuer e Elkington, 1993 [4] |
| V44 | STERNUM | Manubrium length | The longest distance from the midpoint of the manubrium (between the jugular notch and clavicular notch) to the manubriosternal junction. | Place one arm of the caliper at the midpoint of the manubrium (between the jugular notch and clavicular notch) and the other arm at the corresponding point of the junction between the manubrium and the body, keeping the bone perfectly horizontal. | Ramadan et al., 2010 [5] |
| V45 | STERNUM | Body length | The greatest distance between the manubriosternal junction and the mesoxiphoid junction. | Place one arm of the caliper at the middle of the anterior margin of the manubriosternal junction and measure to the inferior point of the mesoxiphoid junction. | Ramadan et al., 2010 [5] |
| V46 | STERNUM | Total length | Combined length of the body and the manubrium, from the midpoint of the manubrium to the mesoxiphoid junction. | Use the landmark points of the midpoint of the manubrium (measurement V44) and the mesoxiphoid junction (measurement V45). | Ramadan et al., 2010 [5] |
| V47 | STERNUM | Manubrium max. width | The maximum width of the manubrium at the level of the most protruding points on the right and left sides. | Place the arms of the caliper at the most lateral protruding points of the manubrium. | Ramadan et al., 2010 [5] |
| V48 | STERNUM | Sup. body width | The latero-lateral width of the sternal body at the level of a line passing through the midpoint of the second and third costal notches on the right and left sides. | Place the arms of the caliper at the midpoints of the lateral edges of the first sternebra of the body. | Ramadan et al., 2010 [5] |
| V49 | STERNUM | Inf. Body width | The latero-lateral width of the sternal body at the level of a line passing through the midpoint of the fourth and fifth costal notches on the right and left sides. | Place the arms of the caliper at the midpoints of the lateral edges of the third sternebra of the body | Ramadan et al., 2010 [5] |
| V50 | 1^ST^ RIB | Max. chord | The maximum distance from the tubercle to the anterior sternal end. | Place the fixed arm of the caliper at the anterior sternal end and the movable arm against the tubercle, until the maximum measurement is achieved. | New measurement |
| V51 | 1^ST^ RIB | Min. chord | The shortest distance from the head to the posterior sternal end. | Place the fixed arm of the caliper against the posterior sternal end and the movable arm against the most anterior portion of the head, to achieve the minimum measurement. | Kubicka e Piontek, 2016 [6] |
| V52 | 4^TH^ RIB | Width | The distance between the upper and lower edges of the fourth rib, just lateral to the costal facet. | Place the caliper arms on the superior and inferior portions of the body of the fourth rib, approximately one centimeter from the costal facet, and rotate the bone to achieve the maximum measurement. | Ramadan et al., 2010 [5] |
| V53 | ATLAS | Sagittal diameter | The maximum distance on the sagittal plane. | Place one arm of the caliper on the most protruding point of the posterior tubercle and the other arm on the most protruding point of the anterior tubercle. | Padovan et al., 2019 [7] |
| V54 | ATLAS | Transverse diameter | The maximum distance on the transverse plane. | Place the arms of the caliper on the most protruding points of the transverse processes on both sides to obtain the maximum measurement. | Padovan et al., 2019 [7] |
| V55 | C2 | Max. sagittal length | The maximum distance on the sagittal plane between the posterior point of the spinous process and the anterior point of the vertebral body. | Place one arm of the caliper on the anterior point of the vertebral body and the other on the posterior point of the spinous process, keeping the bone in its anatomical position. | Wescott, 2000 [8] |
| V56 | C2 | Max. height | The distance between the most protruding superior point of the dens and the inferior point of the anterior margin of the vertebral body. | Place one arm of the caliper on the most superior point of the dens and the other arm on the most inferior point of the anterior margin of the body. Rotate the bone to achieve the maximum measurement. Avoid including any osteophytes on the dens. | Wescott, 2000 [8] |
| V57 | C2 | Max. breadth sup. facets | The maximum distance between the lateral margins of the two superior articular facets. | Place the caliper arms on the most lateral points of the margins of the superior articular facets. | Wescott, 2000 [8] |
| V58 | C7 | Ant. body height | The maximum supero-inferior height of the vertebral body measured on the anterior margin. | Place the caliper arms at the midpoint of the superior and inferior anterior margins of the vertebral body. | Rozendaal et al., 2020 [9] |
| V59 | C7 | Sag. length | The maximum distance on the sagittal plane between the most posterior point of the spinous process and the most anterior point of the vertebral body. | Place one arm of the caliper on the posterior point of the spinous process and the other on the anterior margins of the vertebral body. Maintain the vertebra in its anatomical position, ensuring the caliper is parallel to the superior vertebral body. | Modified from Rozendaal et al., 2020 [9] |
| V60 | C7 | Max. width | The maximum latero-lateral distance on the transverse plane. | Place the caliper arms on the posterior tubercles of the transverse processes on both sides to obtain the maximum measurement. | Rozendaal et al., 2020 [9] |
| V61 | T1 | Ant. body height | The maximum supero-inferior height of the vertebral body measured on the anterior margin. | Place the caliper arms at the midpoint of the superior and inferior anterior margins of the vertebral body. | Garoufi et al., 2020 [10] |
| V62 | T1 | Sag. length | The distance on the sagittal plane between the most posterior point of the spinous process and the most anterior point of the vertebral body | Place one arm of the caliper on the posterior point of the spinous process and the other on the anterior margins of the vertebral body. Maintain the vertebra in its anatomical position, ensuring the caliper is parallel to the superior vertebral body | Modified from Garoufi et al., 2020 [10] |
| V63 | T1 | Width at costal head facets | The maximum transverse distance measured at the costal head facets. | Place the caliper arms on the most laterally protruding points of the costal head facets on both sides. | New measurement |
| V64 | T12 | Ant. body height | The maximum supero-inferior height of the vertebral body measured on the anterior margin. | Place the caliper arms at the midpoint of the superior and inferior anterior margins of the vertebral body. | Garoufi et al., 2020 [10] |
| V65 | T12 | Sag. length | The distance on the sagittal plane between the most posterior point of the spinous process and the most anterior point of the vertebral body | Place one arm of the caliper on the posterior point of the spinous process and the other on the anterior margins of the vertebral body. Maintain the vertebra in its anatomical position, ensuring the caliper is parallel to the superior vertebral body. | Modified from Garoufi et al., 2020 [10] |
| V66 | T12 | Width at costal head facets | The maximum transverse distance measured at the costal head facets. | Place the caliper arms on the most laterally protruding points of the costal head facets on both sides. | New measurement |
| V67 | L1 | Ant. body height | The maximum supero-inferior height of the vertebral body measured on the anterior margin. | Place the caliper arms at the midpoint of the superior and inferior anterior margins of the vertebral body. | Garoufi et al., 2020 [10] |
| V68 | L1 | Sag. length | The distance on the sagittal plane between the most posterior point of the spinous process and the most anterior point of the vertebral body. | Place one arm of the caliper on the posterior point of the spinous process and the other on the anterior margins of the vertebral body. Maintain the vertebra in its anatomical position, ensuring the caliper is parallel to the superior vertebral body. | Modified from Garoufi et al., 2020 [10] |
| V69 | L1 | Max endplate width | The maximum transverse distance of the vertebral body. | Place the caliper arms on the most laterally protruding points of the vertebral body on the right and left sides. The inferior vertebral body generally has the greater measurement | Garoufi et al., 2020 [10] |
| V70 | L5 | Ant. body height | The maximum supero-inferior height of the vertebral body measured on the anterior margin. | Place the caliper arms at the midpoint of the superior and inferior anterior margins of the vertebral body. | Garoufi et al., 2020 [10] |
| V71 | L5 | Sag. length | The distance on the sagittal plane between the most posterior point of the spinous process and the most anterior point of the vertebral body | Place one arm of the caliper on the posterior point of the spinous process and the other on the anterior margins of the vertebral body. Maintain the vertebra in its anatomical position, ensuring the caliper is parallel to the superior vertebral body. | Modified from Garoufi et al., 2020 [10] |
| V72 | L5 | Max endplate width | The maximum transverse distance of the vertebral body. | Place the caliper arms on the most laterally protruding points of the vertebral body on the right and left sides. The inferior vertebral body generally has the greater measurement | Garoufi et al., 2020 [10] |
| V73 | OS COXAE | Max heigth | The maximum distance from the superior point of the iliac crest to the inferior point of the ischial tuberosity. | Place the iliac crest against the vertical axis and adjust the movable upright against the ischial tuberosity, moving the bone to obtain the maximum measurement. | Langley et al., 2016 [1] |
| V74 | OS COXAE | Min. ischium length | The distance from the most medial point on the epiphysis of the ischial tuberosity to the closest point on the acetabular rim. | Place one arm of the caliper against the most infero-medial point of the ischial tuberosity and the other arm against the nearest point of the acetabular rim. | Langley et al., 2016 [1] |
| V75 | OS COXAE | Iliac breadth | The distance between the anterior superior iliac spine and the posterior superior iliac spine. | Place the anterior superior iliac spine against the vertical end board and press the movable upright against the posterior superior iliac spine. Rotate the bone to obtain the maximum measurement. | Langley et al., 2016 [1] |
| V76 | OS COXAE | Min. pubis length | The distance from the symphysion (the uppermost point of the pubic symphysis) to the closest point on the acetabular rim. | Position the fixed arm of the caliper at the symphysion and move the sliding arm until it reaches the nearest point on the acetabular rim. The measurement must be taken directly on the rim, not slightly inside or outside. | Langley et al., 2016 [1] |
| V77 | OS COXAE | Max. I.P ramus length | The distance from the inferior point of the pubic symphysis to the furthest point of the ischial tuberosity. | Place the fixed arm of the caliper on the inferior point of the pubic symphysis and move the sliding arm until it reaches the furthest point on the ischial tuberosity. | Langley et al., 2016 [1] |
| V78 | SACRUM | S1 trans. diameter | The distance between the two most lateral points on the superior articular surface of S1, measured perpendicularly to the sagittal plane that divides the bone in half. | Place the caliper arms on the most lateral points of the superior vertebral body of S1. In the presence of lipping, approximate the original articular margin. The measurement should be perpendicular to the A-P diameter (V79). | Langley et al., 2016 [1] |
| V79 | SACRUM | S1 sagittal diameter | The distance between the most anterior and most posterior points of the superior articular surface of S1, measured along the sagittal plane dividing the bone in half. | Place the caliper arms on the most anterior and posterior points of the superior vertebral body of S1. In the presence of lipping, approximate the original articular margin. The measurement should be perpendicular to the transverse diameter (V78). | Langley et al., 2016 [1] |
| V80 | SACRUM | Anterior height | The distance from the promontory in the sagittal plane dividing the bone in half to the corresponding point on the anterior margin of the sacral apex. | Place one arm of the caliper against the promontory and the other arm against the anterior inferior margin of the fifth sacral vertebra. The measurement is taken in the mid- sagittal plane. Do not consider this measurement if the coccyx is fused or the sacrum exhibits more than five segments. | Langley et al., 2016 [1] |
| V81 | SACRUM | Anterior breadth | The maximum transverse width of the sacrum at the most anterior points of the auricular surfaces | Place the caliper arms on the most anterior projection of the auricular surfaces. If the sacrum is fused with the innominate, the measurement should not be taken. | Langley et al., 2016 [1] |
| V82 | FEMUR | Epicondylar breadth | The distance between the most prominent points of the medial and lateral epicondyles. | Press the arms of the calipers against the most medial point of the medial epicondyle and the most lateral point of the lateral epicondyle. | Langley et al., 2016 [1] |
| V83 | FEMUR | Max. head diameter | The maximum diameter of the femoral head measured at the margin of the articular surface. | Rotate the arms of the caliper around the articular surface of the femoral head to find the maximum diameter. Do not include any arthritic lipping. | Moore-Jansen et al., 1994 [2] |
| V84 | FEMUR | Circumference MS | The circumference of the bone measured at the midshaft. | Determine the midpoint of the shaft using an osteometric board and mark it with a pencil and wrap the tape around the shaft. If the linea aspera is abnormally hypertrophied, take the measurement approximately 10 mm proximal to the midshaft point. | Langley et al., 2016 [1] |
| V85 | FEMUR | Transverse Diameter MS | The transverse diameter measured at the midshaft. | Determine the midpoint of the shaft using an osteometric board and mark it with a pencil. At the determined point, measure the transverse diameter with the caliper. | Moore-Jansen et al., 1994 [2] |
| V86 | FEMUR | Sagittal diameter MS | The antero-posterior diameter measured at the midshaft | Determine the midpoint of the shaft using an osteometric board and mark it with a pencil. At the determined point, measure the antero-posterior diameter with the caliper. | Moore-Jansen et al., 1994 [2] |
| V87 | FEMUR | Transverse subtroch. diameter | The transverse diameter of the proximal shaft at its maximum lateral expansion. | Position the caliper arms on the medial and lateral sides of the shaft just below the lesser trochanter. The transverse diameter should be oriented parallel to the anterior surface of the femoral neck. If this cannot be determined (e.g., in cases of severe torsion), measure approximately 2 cm below the lesser trochanter. | Langley et al., 2016 [1] |
| V88 | FEMUR | Bicondylar length | The distance from the most proximal point of the femoral head to the plane defined by the inferior surfaces of the distal condyles. | Place the femur on the osteometric board with its posterior surface facing down. Press both distal condyles against the vertical end board while positioning the movable upright against the femoral head. | Langley et al., 2016 [1] |
| V89 | FEMUR | Max. length | The distance from the most proximal point of the femoral head to the most distal point on either the lateral or medial condyle | Place the femur on its posterior surface parallel to the long axis of the osteometric board. Position the medial or lateral condyle against the fixed vertical axis and press the movable arm against the femoral head. Adjust the bone to obtain the maximum length. | Langley et al., 2016 [1] |
| V90 | FEMUR | Med. cond. max. length | The distance between the most anterior and most posterior points on the articular surface of the medial condyle. | Place the fixed arm of the caliper on the most anterior point of the articular surface of the medial condyle while pressing the sliding arm against the most posterior point to obtain the maximum measurement. | Langley et al., 2016 [1] |
| V91 | FEMUR | Lat. cond. max. length | The distance between the most anterior and most posterior points on the articular surface of the lateral condyle. | Place the fixed arm of the caliper on the most anterior point of the articular surface of the lateral condyle while pressing the sliding arm against the most posterior point to obtain the maximum measurement. | Langley et al., 2016 [1] |
| V92 | TIBIA | Prox. epiphyseal breadth | The maximum distance between the most prominent margins of the medial and lateral condyles of the proximal epiphysis. | Press the lateral condyle against one arm of the caliper and move the sliding arm against the medial condyle. Tibiae with marked torsion may require rotation to obtain the maximum measurement. | Langley et al., 2016 [1] |
| V93 | TIBIA | Dist. epiphyseal breadth | The distance between the most medial point of the medial malleolus and the lateral surface of the distal epiphysis. | Position the two lateral protrusions against the vertical end board and move the movable upright against the medial malleolus. | Langley et al., 2016 [1] |
| V94 | TIBIA | Nut. for. circumference | The circumference of the tibial shaft measured at the inferior point of the nutrient foramen. | Wrap the tape around the shaft at the level of the inferior point of the nutrient foramen. | Langley et al., 2016 [1] |
| V95 | TIBIA | Nut. for. trans. diameter | The medio-lateral diameter of the tibial shaft measured at the inferior point of the nutrient foramen. | At the level of the inferior point of the nutrient foramen, measure the transverse diameter with the caliper. Keep the bone in its anatomical position. | Moore-Jansen et al., 1994 [2] |
| V96 | TIBIA | Nut. for. AP diameter | The antero-posterior of the tibial shaft measured at the inferior point of the nutrient foramen. | At the level of the inferior point of the nutrient foramen, measure the antero-posterior diameter with the caliper. Keep the bone in its anatomical position. | Moore-Jansen et al., 1994 [2] |
| V97 | TIBIA | Length | The distance from the superior surface of the lateral tibial condyle to the tip of the medial malleolus. | Place the tibia so that its long axis is parallel to the board. Position the lateral condyle against the fixed arm while pressing the movable upright against the medial malleolus. Do not include the intercondylar eminence. If the osteometric board lacks a groove for the intercondylar eminence, adjust accordingly. | Langley et al., 2016 [1] |
| V98 | FIBULA | Max. diameter MS | The maximum diameter of the fibula measured at midshaft. | Determine the midpoint of the shaft using an osteometric board and mark it with a pencil. At the determined point, rotate the bone to find and measure the maximum diameter with the caliper. | Langley et al., 2016 [1] |
| V99 | FIBULA | Max. length | The maximum distance between the superior point of the fibular head and the inferior tip of the lateral malleolus | Place the fibula on the osteometric board. Position the tip of the lateral malleolus against the vertical end board and press the movable upright against the proximal end. Adjust the bone to obtain the maximum length. | Langley et al., 2016 [1] |
| V100 | CALCANEUS | Max. length | The linear distance between the most posteriorly projecting point on the calcaneal tuberosity and the most anterior point on the superior margin of the articular facet for the cuboid, measured in the sagittal plane. | Measure in the sagittal plane by positioning the caliper arms on the most posteriorly projecting point of the calcaneal tuberosity and the most anterior point on the superior margin of the articular facet for the cuboid. | Harris e Case, 2012 [11] |
| V101 | CALCANEUS | Middle breadth | The linear distance between the most lateral projection of the dorsal articular facet and the most medial point of the sustentaculum tali. | Place the caliper arms on the most lateral projection of the dorsal articular facet and the most medial point of the sustentaculum tali. | Harris e Case, 2012 [11] |
| V102 | TALUS | Length | The linear distance from the most posterior point of the trigonal process to the most anterior point of the navicular articular facet. | Position the caliper arms on the most posterior point of the trigonal process and the most anterior point of the navicular articular facet. | Harris e Case, 2012 [11] |
| V103 | TALUS | Breadth | The linear distance from the most lateral point of the lateral process to the medial edge of the talus. | Position the talus with its inferior surface resting on the osteometric board and the medial edge against the vertical end board at two points. Press the movable upright against the most lateral point of the lateral process. | Harris e Case, 2012 [11] |
| V104 | CUBOID | Length | The linear distance from the medial surface to the most proximal point of the cuboid's beak. | Position the cuboid on its medial side with the distal end resting against the fixed arm of the caliper at two contact points. Measure at the most proximal point of the cuboid’s beak. | Harris e Case, 2012 [11] |
| V105 | CUBOID | Breadth | The linear distance from the most medial point on the medial side to the most lateral point on the superior surface of the cuboid. | Position the cuboid on its superior surface with the medial side against the fixed arm of the caliper at two contact points, near the proximal and distal ends. Ensure the tuberosity is oriented upward and measure at the most medial point on the medial side. | Harris e Case, 2012 [11] |
| V106 | NAVICULAR | Length | The linear distance from the proximal face near the superior and inferior ends to the navicular tuberosity. | Position the navicular with its proximal face against the fixed arm of the caliper, ensuring contact at two points along the margins of the proximal facet near the superior and inferior ends. Measure to the navicular tuberosity. | Harris e Case, 2012 [11] |
| V107 | NAVICULAR | Breadth | The linear distance from the medial tuberosity across the proximal face to the most distal point of the navicular. | Position the navicular with its proximal face against the fixed arm of the caliper, ensuring two points of contact across the proximal facet from the medial to the lateral end. The medial tuberosity should rest firmly against the caliper arm. Adjust the sliding arm to measure to the most distal point. | Harris e Case, 2012 [11] |
| V108 | MED CUNEIFORM | Length | The linear distance from the inferior margin of the proximal end to the distal end on the medial surface. | Place the medial cuneiform on its medial surface with the distal end resting against the fixed arm of the caliper at two contact points near the superior and inferior ends. Measure to the inferior margin of the proximal end. | Harris e Case, 2012 [11] |
| V109 | MED CUNEIFORM | Height | The linear distance from the inferior margin to the superior margin of the medial cuneiform, measured along the medial surface. | Place the medial cuneiform on its medial surface with the inferior margin resting against the fixed arm of the caliper at two contact points near the proximal and distal ends. Measure to the superior margin. | Harris e Case, 2012 [11] |
| V110 | INT CUNEIFORM | Length | The linear distance from the proximal end to the distal end along the lateral side of the intermediate cuneiform. | Place the intermediate cuneiform on its lateral side with the proximal end in contact with the fixed arm of the caliper at two points along the superior and inferior margins. Measure to the distal end. | Harris e Case, 2012 [11] |
| V111 | INT CUNEIFORM | Height | The linear distance from the superior margin of the proximal end to the inferior margin on the intermediate cuneiform. | Position the intermediate cuneiform on its proximal end with the superior margin in contact with the fixed arm of the caliper at two points along the medial and lateral edges. Measure to the inferior margin. | Harris e Case, 2012 [11] |
| V112 | LAT CUNEIFORM | Length | The linear distance from the most proximal point of the proximal end to the distal end along the medial side of the lateral cuneiform. | Place the lateral cuneiform on its medial side with the distal end against the fixed arm of the caliper at two contact points near the superior and inferior ends. Measure to the most proximal point of the proximal end. | Harris e Case, 2012 [11] |
| V113 | LAT CUNEIFORM | Height | The linear distance from the superior margin to the inferior margin of the lateral cuneiform, measured along the medial side. | Position the lateral cuneiform on its medial side with the superior margin against the fixed arm of the caliper at two points near the proximal and distal ends. Measure at the inferior margin using the movable arm. | Harris e Case, 2012 [11] |
| V114 | MT1 | Max. length | Maximum distance between the proximal and distal ends of the first metatarsal. | Position one caliper arm against the most protruding point at the proximal end and the other arm against the most protruding point of the distal head. If necessary, rotate the bone on its axis to obtain the maximum measurement. | Case e Ross, 2007 [12] |
| V115 | MT2 | Max. length | Maximum distance between the proximal and distal ends of the second metatarsal. | Position one caliper arm against the most protruding point at the proximal end and the other arm against the most protruding point of the distal head. If necessary, rotate the bone on its axis to obtain the maximum measurement. | Case e Ross, 2007 [12] |
| V116 | MT3 | Max. length | Maximum distance between the proximal and distal ends of the third metatarsal. | Position one caliper arm against the most protruding point at the proximal end and the other arm against the most protruding point of the distal head. If necessary, rotate the bone on its axis to obtain the maximum measurement. | Case e Ross, 2007 [12] |
| V117 | MT4 | Max. length | Maximum distance between the proximal and distal ends of the fourth metatarsal. | Position one caliper arm against the most protruding point at the proximal end and the other arm against the most protruding point of the distal head. If necessary, rotate the bone on its axis to obtain the maximum measurement. | Case e Ross, 2007 [12] |
| V118 | MT5 | Max. length | Maximum distance between the proximal and distal ends of the fifth metatarsal. | Position one caliper arm against the most protruding point at the proximal end and the other arm against the most protruding point of the distal head. If necessary, rotate the bone on its axis to obtain the maximum measurement. | Case e Ross, 2007 [12] |
| V119 | PATELLA | Max length | The greatest linear distance extending from the apex tip to the base of the patella. | Place the fixed arm of the caliper against the most superior point of the bone and move the sliding arm against the corresponding most inferior point. | Knecht et al., 2024 [13] |
| V120 | PATELLA | Max breadth | The greatest linear distance extending from the medial to the lateral borders. | Place the fixed arm of the caliper against the most medial point of the bone and move the sliding arm against the corresponding most lateral point. | Knecht et al., 2024 [13] |
| V121 | PATELLA | Max thickness | The greatest linear distance extending from the anterior to the posterior surface of the patella. | Place the fixed arm of the caliper against the most anterior point of the bone and move the sliding arm against the corresponding most posterior point. | Knecht et al., 2024 [13] |

**References**

1. Langley NR, Jantz LM, Ousley SD, et al (2016) Data collection procedures for forensic skeletal material 2.0. University of Tennessee and Lincoln Memorial University

2. Moore-Jansen PH, Jantz RL, Ousley SD, et al (1994) Data Collection Procedures for Forensic Skeletal Material. Forensic Anthropology Center, Department of Anthropology, University of Tennessee

3. Sulzmann CE, Buckberry JL, Pastor RF (2008) The utility of carpals for sex assessment: a preliminary study. American Journal of Physical Anthropology: The Official Publication of the American Association of Physical Anthropologists 135:252–262

4. Scheuer JL, Elkington NM (1993) Sex determination from metacarpals and the first proximal phalanx. J Forensic Sci 38:769–778

5. Ramadan SU, Türkmen N, Dolgun NA, et al (2010) Sex determination from measurements of the sternum and fourth rib using multislice computed tomography of the chest. Forensic Sci Int 197:120-e1

6. Kubicka AM, Piontek J (2016) Sex estimation from measurements of the first rib in a contemporary Polish population. Int J Legal Med 130:265–272

7. Padovan L, Ulbricht V, Groppo FC, et al (2019) Sexual dimorphism through the study of atlas vertebra in the Brazilian population. J Forensic Dent Sci 11:158

8. Wescott DJ (2000) Sex variation in the second cervical vertebra. J Forensic Sci 45:462–466

9. Rozendaal AS, Scott S, Peckmann TR, Meek S (2020) Estimating sex from the seven cervical vertebrae: an analysis of two European skeletal populations. Forensic Sci Int 306:110072

10. Garoufi N, Bertsatos A, Chovalopoulou M-E, Villa C (2020) Forensic sex estimation using the vertebrae: an evaluation on two European populations. Int J Legal Med 134:2307–2318

11. Harris SM, Case DT (2012) Sexual dimorphism in the tarsal bones: implications for sex determination. J Forensic Sci 57:295–305

12. Case DT, Ross AH (2007) Sex determination from hand and foot bone lengths. J Forensic Sci 52:264–270

13. Knecht S, Morandini P, Biehler-Gomez L, et al (2024) Sex estimation from patellar measurements in a contemporary Italian population: a machine learning approach. Int J Legal Med 1–10
